# Association of childhood trauma with cognitive domains in adult patients with mental disorders and in non-clinical populations: a systematic review

**DOI:** 10.3389/fpsyg.2023.1156415

**Published:** 2023-06-23

**Authors:** Melissa Rosa, Catia Scassellati, Annamaria Cattaneo

**Affiliations:** ^1^Laboratory of Biological Psychiatry, IRCCS Istituto Centro San Giovanni di Dio Fatebenefratelli, Brescia, Italy; ^2^Department of Pharmacological and Biomolecular Sciences, University of Milan, Milan, Italy

**Keywords:** childhood trauma, early life stress, neglect, abuse, cognitive domains, psychotic and mood disorders, non-clinical populations

## Abstract

Although the association between cognitive performances and the onset of psychiatric disorders has been widely investigated, limited research on the role of childhood trauma or early life stress (CT/ELS), and whether this role differs between clinical and non-clinical cohorts is available. This systematic review aims at filling this gap, testing whether the occurrence of CT/ELS and its subtypes are associated with cognitive domains (general cognitive ability, executive functions, working memory, attention, processing speed, verbal/visual memory) in patients with psychiatric disorders and in non-clinical populations. This study followed the PRISMA 2020 guidelines and the Newcastle-Ottawa scale for quality assessment. The search was performed until May 2022. Seventy-four studies were classified as eligible. The graphical representations of the results reported an association between exposure to CT/ELS and worse general cognitive ability, verbal/visual memory, processing speed and attention in patients affected by anxiety, mood and psychotic disorders, and that specific CT/ELS subtypes (physical neglect, physical/sexual abuse) can differentially influence specific cognitive abilities (executive functions, attention, working memory, verbal/visual memory). In non-clinical cohorts we found associations between CT/ELS exposure and impairments in executive functions, processing speed and working memory, while physical neglect was related to general cognitive ability and working memory. Concerning the emotional abuse/neglect subtypes in both populations, the results indicated their involvement in cognitive functioning; however, the few studies conducted are not enough to reach definitive conclusions. These findings suggest an association of CT/ELS with specific cognitive deficits and psychopathology.

## 1. Introduction

It is well known that traumatic experiences can have different long-lasting effects on mental and physical health. According to the Diagnostic and Statistical Manual of Mental Disorders (DSM-IV and DSM-5), traumatic experiences are defined as *exposures to actual or threatened death, serious injury or sexual violence* and include direct trauma exposure, witnessing trauma or learning about traumatic events occurred to friends or relatives. With childhood trauma (CT), we refer to specific adverse psychological conditions encompassing serious adverse childhood experiences (ACEs), such as neglect, physical and sexual abuse, and the National Institute of Mental Health has defined CT as: *“the experience of an event by a child that is emotionally painful or distressful, which often results in lasting mental and physical effects.”*

Several studies have investigated the consequences associated with CT on mental health and cognition in adult individuals. To this regard, young adults with CT history showed an increased risk of developing psychiatric disorders (Teicher et al., 2022), such as schizophrenia spectrum disorder (SZ; Inyang et al., 2022), psychosis (Dvir, 2022), anxiety and major depressive disorder (MDD; McKay et al., 2021), bipolar disorder (BD; McKay et al., 2021), or posttraumatic stress disorder (PTSD; Ressler et al., 2022).

Cognitive functioning refers to multiple mental domains, including memory, attention, information processing and remembering, problem-solving, decision making, and reasoning. Impairments in these cognitive domains have been shown to be associated with several psychiatric disorders (Morozova et al., 2022), and cognitive functions to be genetically correlated with the risk of developing these pathologies (Beck, 2008; Ohi et al., 2018). When looking through a transdiagnostic lens, cognitive dysfunction cuts across disorders. For instance, executive dysfunctions and memory deficits can be observed in patients affected by SZ, MDD, PTSD, and BD (Dere et al., 2010; Czepielewski et al., 2015; Snyder et al., 2015). Moreover, such dysfunctions can persist into remission (Balanzá-Martínez et al., 2005; Semkovska et al., 2019), and be predictors of recurrences (Semkovska, 2021), suggesting that they can be framed independently from psychiatric symptoms. This is the reason why the assessment of cognitive dysfunctions could identify areas of strategic interventions, leading to better outcomes for patients (Millan et al., 2012).

The biological mechanisms underpinning cognitive alterations in psychiatric disorders are complex and involve genetic and epigenetic changes, with variations in the proteomic and metabolomic profiles that, in turn, can be influenced by environmental factors (Morozova et al., 2022). Molecular markers associated with these mechanisms (such as neurotrophic factors, pro-anti-inflammatory cytokines, and markers of oxidative stress) have been found altered in a large proportion of psychiatric patients with cognitive dysfunction (Morozova et al., 2022), suggesting common genetic and metabolic molecular mechanisms across different diagnoses of psychiatric disorders. Therefore, the identification of genetic, epigenetic, neurotrophic, inflammatory, and oxidative biological markers of cognitive decline may improve the understanding of the pathogenesis of psychiatric disorders and suggest diagnostic and prognostic strategies, opening the way to the design of more effective interventions (Morozova et al., 2022).

Importantly, several studies have shown that some cognitive biases and neurocognitive domains can mediate the relationship between adversity (CT or early life stress, ELS) and the onset of psychotic disorders (Mansueto et al., 2019; Alameda et al., 2020; Rodriguez et al., 2021). Howes and Murray developed a socio-developmental-cognitive model, supporting an integrated description of how the social environment can lead to psychosis through neurobiological changes in the brain along with cognitive bias (Howes et al., 2014). Results from a previous meta-analysis demonstrated a significant negative association between overall cognition and childhood adversity, observed in 3,315 individuals with a psychotic illness (Vargas et al., 2019). When analyzing specific subdomains of cognition, a negative, albeit modest, association was observed between CT and working memory (WM). The authors concluded that a detailed mapping of the different types of childhood adversities, timing of the trauma and severity of exposure is important to move this research area forward.

With respect to the general population, limited evidence supports the effect of ACEs on cognition (Zielinski, 2009; van Os et al., 2017), as only few studies are available to date on this topic. For instance, Majer et al. (2010) showed that infants and toddlers with a history of abuse, neglected, or exposed to multiple medical and surgical procedures often exhibited deficits in cognitive skills. A recent review (Aafjes-van Doorn et al., 2020) has shown that cognitive features can mediate the link between early trauma and later psychopathology (depression, anxiety, eating disorders, and PTSD) and can also represent potential intervention targets for individuals who, although exposed to CT/ELS, have not yet developed psychopathology.

The implications of increased incidence of future cognitive decline and psychiatric disorders in thus consolidated. However, limited research has been dedicated to the potential role of CT/ELS on cognitive performance in clinical and non-clinical adults. To fill the gap in this field, we structured a systematic review delineating a complex and wide umbrella under which we reported and described studies including different types of childhood adversity, different cognitive domains, different psychiatric diseases, and different targets (clinical and non-clinical populations). In particular, we aim at describing and discussing the existing evidence on the association between specific CT events and ELS profiles (emotional, physical and sexual abuse, as well as emotional and physical neglect) with specific cognitive domains (general cognitive ability, GCA; executive functions, EFs; WM, attention and processing speed, PS; verbal and visual memory) in adults affected by different psychiatric disorders and also in non-clinical populations.

## 2. Methods

The search was conducted in accordance with the Preferred Reporting Items for Systematic reviews and Meta-Analyses (PRISMA) 2020 guidelines (Page et al., 2021). The PRISMA checklist was reported in Supplementary material.

### 2.1. Literature search strategy

From the inception to May 2022, the following major public scientific databases and platforms were searched: PubMed, Embase and PsycInfo. Starting with original papers, we assessed the association between traumatic childhood events, general cognitive performance, and psychiatric disorders at adulthood. The search was performed using keywords and/or a medical subject heading (MeSH) strategy. The keywords “cognit*,” “childhood trauma,” “psychiatry” and “healthy” were applied to get the related literature. The MeSH strategy was based on the specific terms/syntax (Table 1).

**Table 1:** Database search results using regular keywords and MeSH strategy.

Keywords	Database	Initial search results
((((childhood abuse[Title/Abstract] OR childhood sexual [Title/Abstract] OR childhood neglect[Title/Abstract] OR childhood trauma[Title/Abstract] OR childhood maltreatment[Title/Abstract] OR childhood adversity [Title/Abstract] OR early life stress[Title/Abstract] OR early life trauma [Title/Abstract])) AND (cognit*[Title/Abstract] OR neurocognit*[Title/Abstract] OR memory[Title/Abstract]))) AND English [Language] Filtro “Human”	Pudmed	1,379
(‘childhood abuse’:ab,ti OR ‘childhood sexual’:ab,ti OR ‘childhood neglect’:ab,ti OR ‘childhood trauma’:ab,ti OR ‘childhood maltreatment’:ab,ti OR ‘childhood adversity’:ab,ti OR ‘early life stress’:ab,ti OR ‘early life trauma’:ab,ti) AND (cognit*:ab,ti OR neurocognit*:ab,ti OR memory) AND [embase]/lim AND [humans]/lim AND ([article]/lim OR [article in press]/lim) AND [English]/lim	Embase	1,004
(abstract: childhood abuse OR abstract: childhood sexual OR abstract: childhood neglect OR abstract: childhood trauma OR abstract: childhood maltreatment OR abstract: childhood adversity OR abstract: early life stress OR abstract: early life trauma) AND (abstract: cognit* OR abstract: neurocognit* OR abstract: memory) AND Any Field: Population Group: Human	PsycInfo	134

MeSH: Medical subject heading search strategy.

The screening was performed independently by two authors (MR and CS) who were unaware of each other’s decisions. Disagreements were resolved by reaching a consensus.

### 2.2. Inclusion/exclusion criteria

A PRISMA flow diagram 2020 was used to show the study’s inclusion and exclusion of articles found in the databases. We included studies that involved: (1) adults (mean age ≥ 18 years); (2) subjects affected or not by psychiatric disorders; (3) diagnosis performed by standardized, validated diagnostic scales according to the DSM IV, DSM-5 or the International Classification of Diseases (ICD-9/10); (4) subjects exposed to CT/ELS; (5) CT/ELS assessments in general and in relation to sexual abuse, physical abuse, neglect, or emotional/psychological abuse performed by using self-report tests, other reports or official records; (6) an assessment of higher cognition functions (GCA; EFs; WM; attention; PS; verbal and visual memory) performed by standardized neuropsychological tests; (7) a study design as longitudinal, cross-sectional, or case–control approach. By “longitudinal” we meant those studies that included, at baseline, CT/ELS exposed adolescent/young adult subjects who were valued for cognition and psychological symptoms. Then, they were re-tested according to clinical and cognitive assessment at different time-points or follow up during adulthood/elderly and that the results were compared to CT/ELS data collected at baseline (retrospective data). In this specific case, we included in our analyses the data obtained starting from the last time-point.

We excluded studies that meet the following criteria: (1) focusing on social cognition (e.g., Theory of the Mind, emotional regulation, coping strategy), recovery traumatic memory (e.g., amnesia, false memory, autobiographical memory), neurological diseases (e. g., injury trauma, neurodegenerative diseases) and patients with any disease that could influence cognitive processes (e.g., dementia, HIV, diabetes, drugs, and alcohol), or affected by personality disorders; (2) performing in children/adolescent cohorts or based on preclinical, biological, neuroimaging, neurophysiological and electroencephalogram research; (3) review articles, books, and articles not published in the English language. There was no restriction on year of publication.

### 2.3. Data extraction

MR and CS independently extracted the following data: first author and year of publication, study design (cross-sectional, case–control and cohort longitudinal study), sample size (N), % of female, mean age, years of education, ethnicity, different kinds of pathologies, studies performed on non-clinical sample, CT/ELS assessment and type of trauma, cognitive assessment, type of cognition domain and psychopathology symptoms.

### 2.4. Quality assessment and strength of evidence

The Newcastle-Ottawa quality assessment scale was used to assess methodological quality and risk of bias (Wells et al., 2014). In brief, each study is rated on three broad criteria: (1) selection of the study groups; (2) comparability of the groups; and (3) the ascertainment of the exposure or outcome of interest. A score ≥ 7 for case–control and cohort studies, and ≥ 6 for cross-sectional studies was considered indicative of “good” quality and bias control. Two reviewers independently applied the device, and discrepancies were resolved through discussion with the third reviewer. As this is an under-researched area, all studies were included regardless of quality rating.

### 2.5. Childhood trauma assessment

In this review, we employed a general definition of physical, sexual, and emotional abuse as an act causing injury or trauma in the respective domains.

All CT/ELS tests employed in the selected studies are described in the Supplementary material. Here below, we reported the most used questionnaires across the studies.

Several tests, questionnaires, and checklist are available to measure CT/ELS. The most used test to evaluate the occurrence of CT is the Childhood Trauma Questionnaire (CTQ/CTQ-Short form; Bernstein et al., 1994, 2003). The CTQ is a self-reported questionnaire designed to assess CT occurring before the age of 18 years. It includes 70 items (28 in the short form) to evaluate five categories of CT experience, including emotional, physical, and sexual abuse, and emotional and physical neglect.

The Childhood Experiences of Care and Abuse (CECA-Q), the Childhood Abuse Questionnaire (CAQ), the Early Trauma Inventory (ETI/ETI-Short form), the Stressful life Events Screening Questionnaire (SLESQ) and the Childhood Traumatic Events Scale (CTES) are other tests that can assess childhood and adolescent experience of neglect and abuse. These questionnaires evaluate multiple aspects of caregiving experiences, including physical, sexual, emotional abuse and maltreatment experience before the age of 17 (Pennebaker and Susman, 1988; Bifulco et al., 1994, 1997; Goodman et al., 1998; Bremner et al., 2000; Rosenman and Rodgers, 2004).

Other studies employed the Traumatic Antecedents Questionnaire (TAQ), another self-report instrument available that gathers information about traumatic lifetime experiences, evaluating the exposure to traumatic events across four age periods, young childhood (0–6 years), school age childhood (7–12), adolescence (13–18) and adulthood (Park et al., 2020).

### 2.6. Cognitive domains assessment

Based on literature research, we analyzed studies that assessed the general CGA, but also studies reporting data on specific cognitive subdomains such as EFs, WM, attention, PS, verbal and visual memory. All the neuropsychological batteries used in the selected studies are described in the Supplementary material. Here below, we labelled the most used tests across the studies.

#### 2.6.1. General cognitive ability

GCA refers to a trait-like ability associated with performance across different cognitive tasks and it includes intelligent quotient (IQ). This ability can be measured by using multiple cognitive tests, such as the Wechsler Adult Intelligence Scale (WAIS), the Wechsler Abbreviated Scale of Intelligence (WASI), the Repeatable Battery for the Assessment of Neuropsychological Status (RBANS), the National Adult Reading Test (NART), the Cambridge Neuropsychological test automated Battery (CANTAB), the Wechsler Test of Adult Reading (WTAR). The Mini-Mental State Exam (MMSE) and the Montreal Cognitive assessment (MoCa) are two other forms of testing widely used to assess cognitive functioning among the elderly and they include tests of orientation, attention, memory, language, and visual–spatial skills (Korsnes, 2020).

#### 2.6.2. Executive functions

EFs represent a set of cognitive skills that involve top-down control processes elicited in the planning, organizing, and monitoring of complex, goal-directed behaviors. EFs include high-order cognitive abilities such as inhibitory control, cognitive flexibility, planning, reasoning, and problem solving (Cristofori et al., 2019). It also includes the WM, that is here described in a dedicated section (see below “*Working Memory”* paragraph).

EFs can be measured by different tests that cover one or more specific components. For instance, the Wisconsin Card Sorting Test (WCST) is one of the most widely used complex EFs tasks available for various populations. The participants are expected to accurately sort each response card with one of four stimulus cards through the feedback (right or wrong) given to them according to a rule based on three possible sorting categories shape, number and color. Another available test is the Behavior Rating Inventory of Executive Function-adult version (BRIEF-A), a standardized measure with self-and informant-report versions that assess perception of behavioral and emotional manifestations of executive dysfunction in daily life.

Verbal fluency is sometimes considered an EF ability (Gustavson et al., 2019) and it is often assessed by measuring phonological and semantic fluency (WAIS-R subtest; Controlled Oral Word Association Test, COWAT). In the semantic fluency tasks, subjects are required to generate words belonging to a category (e.g., animals) within a limited time window, whereas in phonemic fluency subjects must generate words starting with a given letter.

#### 2.6.3. Working memory

Based on the involvement of specific cognitive domains, different definitions of WM have been proposed over the years. Progressively, a consensus was reached acknowledging that WM is extensively involved in goal-directed behaviors in which the retainment and manipulation of specific information is crucial to ensure successful task execution. The multicomponent WM model proposed by Baddeley (2012) described well the concept of WM.

This cognitive ability can be assessed by different tests, such as the Letter–Number Span Test (LNS), digit span tasks, the Operation Span Task (OSPAN), the WAIS-III or Wechsler Memory Scale (WMS-III/WMS-R), or the Response Shifting Task (RST).

#### 2.6.4. Attention and processing speed

Attention is the process of selecting for active processing specific aspects of the physical environment (e.g., objects) or ideas stored in the memory (Spielberger, 2004). PS is an individual cognitive ability measuring the time required by each person to understand or react to specific cognitive tasks (Strauss et al., 2006).

The assessment of attention and PS can be performed by using different batteries including the Digit Span-Forward (Wechsler, 1981) which is a measure of immediate attention and rote recall, whereas the Trail Making Test – Part A-B (TMT-A, B; Reitan, 1958) is a test of speeded attention, mental tracking and visual search. Participants are required to connect a series of circles containing numbers randomly arranged in a spatial array. Both time-to-completion in seconds, as well as error rate, indicate an individual’s level of attention. The Continuous Performance Test (CPT/CPT-R/CPT-II/CPT-HQ) and the Sustained Attention to Response Task (SART) are additional tests which specifically assess the sustained attention. Other batteries include the California Computerized Assessment Package (CALCAP) that is used for sequential reaction time and choice reaction time; and the Paced Auditory Serial Addition Test (PASAT) that assesses auditory information PS and flexibility, as well as calculation ability.

#### 2.6.5. Verbal and visual memory

Memory refers to the psychological processes of acquiring, storing, retaining, and later retrieving information. There are three major processes involved in memory: encoding, storage, and retrieval. Encoding refers to the processing of information to be stored. For example, you can encode a list of spelling words by reviewing them multiple times. The repetition of information leads to the consolidation, or strengthening of its representation while it is stored (Banich and Compton, 2018).

Memory can be evaluated through a series of tests and subtests that allow to investigate the different components, such as verbal and visual memory, as well as the ability to remember information immediately after it has been presented (short-term memory) and after a specified delay (long-term memory).

There are different tests to assess verbal memory domain; for instance, the Rey Auditory Verbal Learning Test (RAVLT) or the California Verbal Learning Test (CVLT) and a subtest of the WMS. In all of them, participants are invited to recall a list of words or a short story, both immediately and after a time delay. The Rey–Osterrieth Complex Figure Test (ROCFT) and the Taylor Complex Figure Test (TCFT) are generally exploited to measure visuospatial memory reproduction and memory recall of specific visual design. Other tests are the Verbal Learning and Memory Test (VLMT), the Hopkins Verbal Learning Test-Revised (HVLT-R), and the RBANS subtest that is used for assessing the verbal, non-verbal and logical memory.

### 2.7. Graphical representations

A set of pie charts was used to graphically summarize the results of the studies included in this review. All of the hypothesized analysis plans clashed with the heterogeneity of the studies in terms of considered populations, outcome measures and scales used to evaluate the exposure of interest (CT or cognitive domains). Such heterogeneity directly results from the explorative purpose of our manuscript, as we aimed at systematically review the broad family of studies evaluating any association between CT exposure and cognitive functioning rather than focusing on a narrower slice of the literature using specific cognitive assessment and CT exposure scales. In this context, we believe the pie charts to do a good job in summarizing the key results of the literature as, with a single glance, it is possible to grasp both how many studies have focused on a specific association between a CT type and cognitive function (by looking at the size of the pies, if any) and the results of those studies (by looking at the sizes of the slices. We first divided the results of the studies according to the target population: (1) individuals with psychotic, mood and anxiety disorders; (2) individuals with a psychosis diagnosis; (3) individuals with mood and anxiety disorders; and (4) non-clinical populations. Then the results were divided according to the type of CT (general CT/ELS, physical, sexual or emotional abuse and emotional or physical neglect) and cognitive ability (GCA, EFs, WM, attention, PS, verbal/visual memory). Moreover, for each pair of CT type and cognitive ability (36 pairs, corresponding to 6 types of CT times 6 cognitive abilities), we considered the studies that included data on the association between history of CT and cognition in that specific context and computed the proportion of the studies that found no association, lower and higher performance in the CT group compared to no CT group. The resulting proportions were represented on a grid of 36 pie charts, whose area was made proportional to the number of studies considered in each pair of CT type and cognitive ability. Analyses were performed with R, version 3.6.0.

## 3. Results

**Figure 1:**
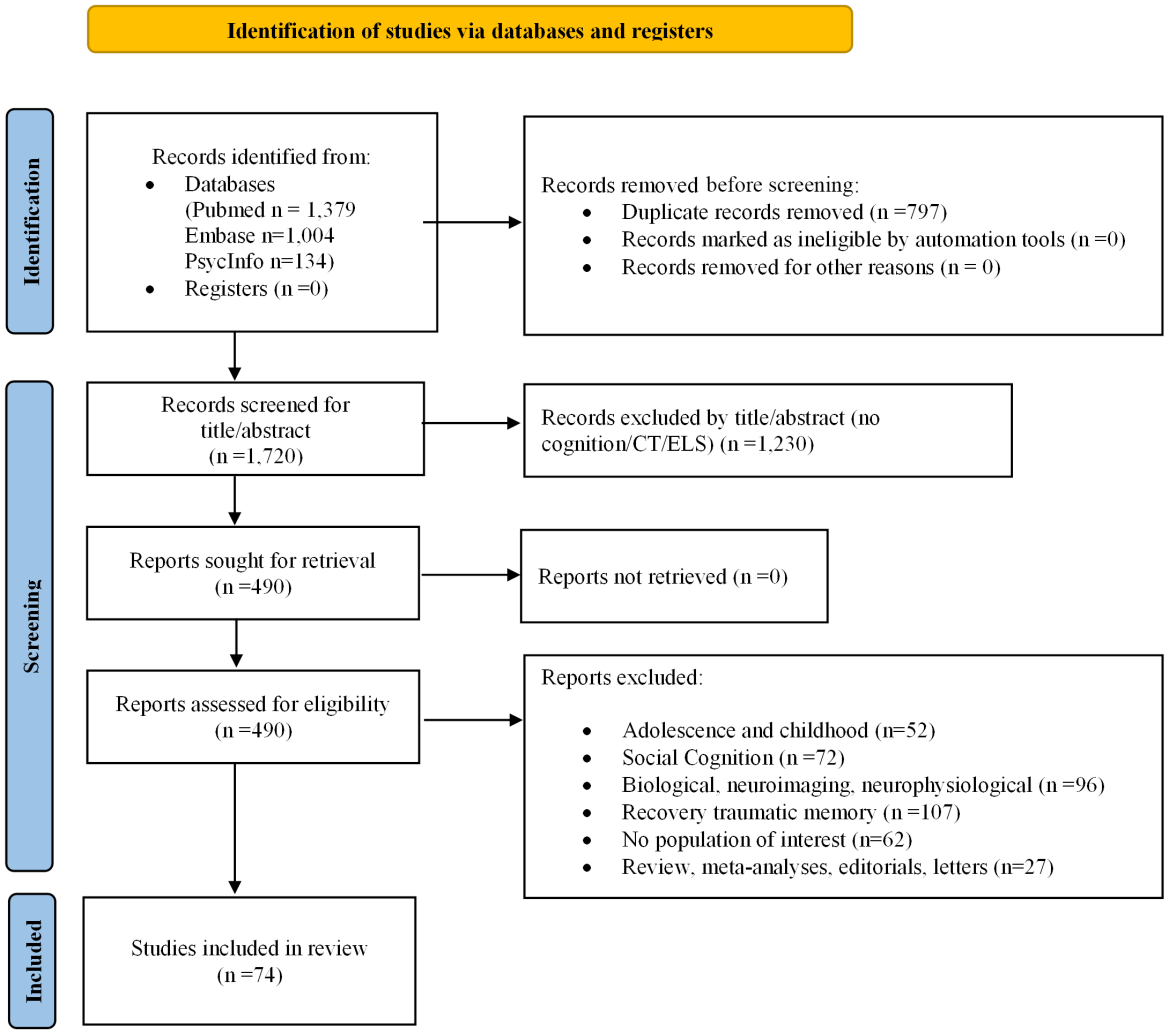
PRISMA flow chart.

The selection and the progressive removal of the identified articles are summarized in the PRISMA flowchart provided in Figure 1.

Our database query retrieved 1,379 (Pubmed); 1,004 (Embase); 134 (PsycInfo) records. Before the screening, we removed 797 duplicates. Then, 490 reports were identified and assessed for eligibility. After further screening, 74 publications investigating CT/ELS, cognitive domains and psychotic, mood and anxiety mental disorders were assessed for eligibility via the analysis of their full-text version. Included in the 74 selected findings, we found 15 and 12 studies related to SZ and psychotic disorders respectively; 8 studies for BD; 9 for MDD; 9 for PTSD; and 2 for GAD (generalized anxiety disorder)/anxiety. We did not find studies related to obsessive–compulsive disorder, somatic disorders, dissociative disorders, eating disorders, and conduct disorders. We selected 24 studies that were performed in non-clinical populations. Concerning the cognitive domains classification, we selected 47 studies on GCA; 40 on EFs; 34 on WM; 36 on attention and PS; and 50 on verbal/visual memory.

### 3.1. Study characteristics

The study characteristics have been summarized in the Supplementary material: Supplementary Table S1 (schizophrenia spectrum and other psychotic disorders), Supplementary Table S2 (mood and anxiety disorders: BD, MDD, PTSD, GAD), Supplementary Table S3 (non-clinical samples). These tables describe each study specifying the first author and the year of publication, study design, sample source, sample size (*N* = number of patients and controls), % of females, mean age, mean years of education, ethnicity, psychiatric disorders, CT/ELS domains, trauma measures, cognitive domains, cognitive measures, psychopathology symptoms (Supplementary Table S3).

All the studies reported data on adult individuals, with most of the studies (36) having subjects in the age range of 18–35 years old and only 5 studies were focused on the elderly, i.e., age ≥ 65. Regarding the design of the study, 29 were case–control studies, 37 were cross-sectional, whereas 8 were longitudinal studies (see Supplementary Tables S4–S6).

Out the 74 selected studies, 50 were conducted on patients affected by psychotic, mood and anxiety disorders (SZ, psychosis, MDD, BD, PTSD, anxiety/GAD; Supplementary Tables S1, S2). In particular, 13 studies included patients affected by SZ (Lysaker et al., 2001, 2002; Schenkel et al., 2005; Shannon et al., 2011; McCabe et al., 2012; Kelly et al., 2016; Li et al., 2017; Ruby et al., 2017; Kilian et al., 2018; Mørkved et al., 2020; Wells et al., 2020; De-Nardin et al., 2021; Kasznia et al., 2021), and 12 were conducted in patients with ultra-high risk of psychosis (Üçok et al., 2015; Velikonja et al., 2019, 2021) or in patients with first-episode psychosis, FEP (Aas et al., 2011, 2012b; Sideli et al., 2014; Ayesa-Arriola et al., 2020) or patients with psychosis (Garcia et al., 2016; van Os et al., 2017; Mansueto et al., 2018, 2019; Schalinski et al., 2018). Four studies included patients affected by BD (Bücker et al., 2013; Jiménez et al., 2017; Martins et al., 2019; Hsieh et al., 2020), 6 studies were conducted in patients with MDD (Gould et al., 2012; Dannehl et al., 2017; Saleh et al., 2017; Kaczmarczyk et al., 2018; Chakrabarty et al., 2020; Goltermann et al., 2021), while 9 studies included subjects with PTSD (Bremner et al., 1995, 2004; Stein et al., 1999; Pederson et al., 2004; Burri et al., 2013; Nikulina and Widom, 2013; Rivera-Vélez et al., 2014; Nakayama et al., 2020; Lantrip et al., 2023). Only one study considered subjects affected by GAD (Petkus et al., 2018). Moreover, 5 studies included mixed diagnosis: 2 involved patients with SZ and BD (Aas et al., 2012a; Quidé et al., 2018), 2 were performed in patients with MDD and BD (Savitz et al., 2008; Poletti et al., 2017) and one study on patients with depressive symptoms and anxiety (Tjoelker et al., 2022).

A total of 24 studies included non-clinical populations (Supplementary Table S3).

### 3.2. Childhood trauma assessment

When selecting studies according to CT/ELS assessment, we found that 36 works applied the CTQ/CTQ-Short form questionnaire to evaluate the occurrence of CT (Supplementary Tables S1–S3); 5 used the CECA-Q to evaluate the abuse and caregiver neglect (Aas et al., 2011, 2012b; Sideli et al., 2014; Chakrabarty et al., 2020; Kasznia et al., 2021), while 3 utilized the CAQ (Lysaker et al., 2002; McCabe et al., 2012; Wells et al., 2020), and 3 the ETI to describe the specific details of the abuse (Bremner et al., 1995, 2004; Ruby et al., 2017). Only one study used the Early Trauma Inventory Self-Report Short Form (ETISR-SF; Petkus et al., 2018).

To assess the nature of the stressors, other studies (Feeney et al., 2013; Cromheeke et al., 2014; Saleh et al., 2017) employed the SLESQ. Navalta et al. (2006) utilized the TAQ for evaluating only the sexual abuse in college women. One study implemented these three different childhood abuse scales: the Lifetime Report of Physical Abuse (LPAA), the Lifetime Report of Psychological Abuse (PALA), and the Lifetime Report of Sexual Abuse (SALA) to assess a young adult’s self-reported history of lifetime abuse and harsh parenting (Mark et al., 2019). Goldberg et al. (2013) and Halpin et al. (2022) used the Adverse Childhood Experiences Questionnaire (ACEQ) to assess parental loss, bullying and experiences of abuse and of neglect. Another study (Ayesa-Arriola et al., 2020) employed the Childhood Trauma Events Scale (CTES), a brief survey of different types of CT experienced; Poletti et al. (2017) evaluated the parental neglect, such as family conflict and malnutrition, with the Risky Families Questionnaire (RFQ). Schalinski et al. (2018) utilized the Maltreatment and Abuse Chronology of Exposure scale (MACE). One study (Roberts et al., 2022) employed the physical/emotional abuse subscales of CTQ along with the Sexual Maltreatment Scale of the Conflict Tactics Scales (sexual abuse subscale). A further study (Rivera-Vélez et al., 2014) applied the self-report Psychological Clinical Science Accreditation System (PCSASS) for studying the abuse.

Eleven studies recorded information on possible history of trauma with *ad-hoc* clinical interviews or trauma checklists, based on the classic interviews of the DSM-5 (Stein et al., 1999; Lysaker et al., 2001; Schenkel et al., 2005; Ritchie et al., 2011; Burri et al., 2013; Korten et al., 2014; Kobayashi et al., 2020; Hawkins et al., 2021; Lewis et al., 2021; Velikonja et al., 2021; Tjoelker et al., 2022), two studies used information reported by the processed case for abuse (Perez and Widom, 1994; Nikulina and Widom, 2013), and one recovered information described in the national archives (Pesonen et al., 2013).

### 3.3. Cognitive domains assessment

#### 3.3.1. General cognitive ability

Here, we described all the studies that analyzed CGA abilities, as showed in the Supplementary material (Supplementary Tables S1–S3). Most of the studies (*N* = 24 in psychiatric, *N* = 4 in non-clinical samples) assessed GCA by averaging the WASI or WAIS/WAIS-R-III–IV/short-form. Seven studies performed in psychiatric patients used the RBANS (Kelly et al., 2016; Li et al., 2017; Petkus et al., 2018; Quidé et al., 2018; Nakayama et al., 2020; Wells et al., 2020; Kasznia et al., 2021). In 3 studies, the premorbid and current IQ were assessed using the NART (Shannon et al., 2011; Bücker et al., 2013; Sideli et al., 2014), whereas one study (Ritchie et al., 2011) used the same battery applied to non-clinical populations. Two papers employed the CANTAB tested in psychiatric patients (Gould et al., 2012; Bücker et al., 2013), 2 the WTAR (McCabe et al., 2012; Wells et al., 2020), whereas 3 in non-clinical samples (Majer et al., 2010; Syal et al., 2014; Lewis et al., 2021). In psychiatric subjects, 2 works exploited the Word Learning Task (WLT; Mansueto et al., 2018, 2019), whereas 5 employed a specific neuropsychological battery for SZ (Garcia et al., 2016; Poletti et al., 2017; Kilian et al., 2018; Schalinski et al., 2018; De-Nardin et al., 2021). GCA in older psychiatric people was measured by the MMSE in 2 studies (Dannehl et al., 2017; Petkus et al., 2018), and one study used both the MMSE and a structured interview for the diagnosis of dementia (SIDAM; Burri et al., 2013), whereas in non-clinical populations, one study implemented both the MMSE and the MoCa (Feeney et al., 2013) or only the MMSE (Korten et al., 2014). Finally, the non-verbal and fluid intelligence were evaluated with the Raven’s Matrices (RSMP) in 2 studies (Aas et al., 2011, 2012b) performed in psychiatric samples.

#### 3.3.2. Executive functions

The aspects of EFs, i.e., control initiation, cognitive flexibility and decision making, were measured through several tests (Supplementary Tables S1–S3). In psychiatric patients, the WCST was utilized in 9 studies (Lysaker et al., 2001, 2002; Savitz et al., 2008; Üçok et al., 2015; Jiménez et al., 2017; Poletti et al., 2017; Ruby et al., 2017; Hsieh et al., 2020; Mørkved et al., 2020), with one study using a modified version of this battery (Dannehl et al., 2017); whereas one study was performed in non-clinical populations (Mark et al., 2019). The Stroop test was implemented in 6 studies conducted in psychiatric samples (Savitz et al., 2008; Üçok et al., 2015; Jiménez et al., 2017; Saleh et al., 2017; Mørkved et al., 2020; Tjoelker et al., 2022). The BRIEF-A was applied to psychiatric patients in one study (Lantrip et al., 2023), whereas it was applied to non-clinical populations in two studies (Daly et al., 2017; Letkiewicz et al., 2021). The EFs were also tested through tasks measuring phonological and semantic fluency (WAIS-R subtest, COWAT) in 6 studies (Schenkel et al., 2005; Savitz et al., 2008; Jiménez et al., 2017; Ruby et al., 2017; Saleh et al., 2017; Quidé et al., 2018) conducted in psychiatric samples. Other studies performed in non-clinical populations employed a subtest of neuropsychological batteries (Majer et al., 2010; Pluck et al., 2011; Feeney et al., 2013; Lewis et al., 2021; D’Amico et al., 2022; Halpin et al., 2022; Roberts et al., 2022).

#### 3.3.3. Working memory

The literature research showed the use of the most common batteries to assess WM abilities (Supplementary Tables S1–S3). In psychiatric samples, the WM performance was assessed in 27 studies by using these tests or subtests: LNS; digit span tasks; OSPAN; WAIS-III or WMS subtests; RST. The same tests were used in non-clinical populations (Goldberg et al., 2013; Mark et al., 2019; Hawkins et al., 2021), with the only exception being for Roberts et al. (2022) who valuated WM tasks by using the Cogstate Brief Battery (CBB).

#### 3.3.4. Attention and processing speed

In psychiatric patients, attention and PS domains were assessed in 15 studies by the TMT A-B (Aas et al., 2011, 2012b; Bücker et al., 2013; Nikulina and Widom, 2013; Sideli et al., 2014; Dannehl et al., 2017; Jiménez et al., 2017; Ruby et al., 2017; Saleh et al., 2017; Kaczmarczyk et al., 2018; Velikonja et al., 2019, 2021; Ayesa-Arriola et al., 2020; Mørkved et al., 2020; Goltermann et al., 2021), whereas 2 studies employed this battery in non-clinical populations (Ritchie et al., 2011; Halpin et al., 2022). Seven studies used different versions of the CPT tested in psychiatric subjects (Lysaker et al., 2002; Üçok et al., 2015; Jiménez et al., 2017; Mansueto et al., 2018, 2019; Ayesa-Arriola et al., 2020; Velikonja et al., 2021). Only one study utilized CPT administrated to non-clinical samples (Mark et al., 2019). Two studies employed the PASAT (Velikonja et al., 2019) or the CALCAP (Mørkved et al., 2020) in psychiatric patients. The other studies that assessed the attention applied a subtest of general batteries such as the WAIS-R and the WAIS-III digit symbol subtests (Aas et al., 2011; Ayesa-Arriola et al., 2020). In non-clinical populations, SART was tested only in one study (Feeney et al., 2013; Supplementary Tables S1–S3).

#### 3.3.5. Verbal and visual memory

To assess the visuo-spatial memory reproduction and memory recall, 5 studies performed in psychiatric samples (Savitz et al., 2008; Jiménez et al., 2017; Kaczmarczyk et al., 2018; Ayesa-Arriola et al., 2020; Mørkved et al., 2020) used the ROCFT and, of these, one study performed also the TCFT (Kaczmarczyk et al., 2018). The other 20 studies implemented a specific memory subtest of a general memory battery test: 13 studies employed the WMS-subtest, whereas 7 used the RBANS-subtest. To assess verbal memory domain, 21 studies utilized the RAVLT, CVLT, VLMT or HVLT-R. In non-clinical samples, 2 studies performed the RAVLT (Hawkins et al., 2021; Halpin et al., 2022), whereas the VLMT was used by Terock et al. (2020), and CBB by Roberts et al. (2022) (Supplementary Tables S1–S3).

### 3.4. CT/ELS exposure and specific cognitive domains in subjects affected by schizophrenia spectrum disorders and psychosis

Table 2 shows the main results on the association between CT/ELS (and their relative subtypes) and specific cognitive profiles in patients affected by SZ, FEP and psychotic disorders.

**Table 2:** Summary of results from studies investigating childhood trauma (CT)/early life stress (ELS) and cognitive functions in schizophrenia, first episode and psychotic disorders.

Cognitive domains	Trauma and subtypes	Psychotic disorders	Results	References
General cognitive ability	General CT/ELS	SZ	LW	McCabe et al. (2012)
		SZ	LW	De-Nardin et al. (2021)
		SZ	LW	Schenkel et al. (2005)
		SZ	LW	Kasznia et al. (2021)
		SZ	NO	Ruby et al. (2017)
		SZ	NO	Kelly et al. (2016)
		SZ	NO	Lysaker et al. (2002)
		SZ	NO	Wells et al. (2020)
		SZ	NO	Lysaker et al. (2001)
		FEP	LW	Aas et al. (2012b)
		FEP	LW	Aas et al. (2011)
		FEP	NO	Sideli et al. (2014)
		FEP	NO	van Os et al. (2017)
		PSY	NO	Üçok et al. (2015)
		PSY	NO	Garcia et al. (2016)
		PSY	LW	Schalinski et al. (2018)
	Physical abuse	SZ	NO	Kilian et al. (2018)
		SZ	LW	Aas et al. (2012a)
		PSY	NO	Velikonja et al. (2021)
	Sexual abuse	SZ	NO	Kilian et al. (2018)
		SZ	NO	Aas et al. (2012a)
		PSY	NO	Üçok et al. (2015)
		PSY	NO	Velikonja et al. (2021)
	Emotional abuse	SZ	NO	Kilian et al. (2018)
		PSY	NO	Üçok et al. (2015)
		PSY	HG/NO	Velikonja et al. (2021)
	Emotional neglect	SZ	LW	Kilian et al. (2018)
		PSY	NO	Üçok et al. (2015)
		PSY	HG/NO	Velikonja et al. (2021)
	Physical neglect	SZ	LW	Kilian et al. (2018)
		SZ	NO	Aas et al. (2012a)
Executive functions	General CT/ELS	SZ	NO	Quidé et al. (2018)
		SZ	NO	Lysaker et al. (2002)
		SZ	NO	Schenkel et al. (2005)
		FEP	LW	Aas et al. (2012b)
		FEP	LW	Aas et al. (2011)
		PSY	LW	Velikonja et al. (2019)
	Physical abuse	SZ	LW	Li et al. (2017)
		PSY	LW	Üçok et al. (2015)
		SZ	NO	Aas et al. (2012a)
	Sexual abuse	SZ	LW	Lysaker et al. (2001)
		SZ	LW	Li et al. (2017)
		SZ	NO	Aas et al. (2012a)
	Emotional neglect	SZ	NO	Aas et al. (2012a)
	Physical neglect	SZ	LW	Li et al. (2017)
		SZ	LW	Aas et al. (2012a)
Working memory	General CT/ELS	SZ	NO	Quidé et al. (2018)
		SZ	LW	Shannon et al. (2011)
		FEP	LW	Aas et al. (2011)
		PSY	LW	Velikonja et al. (2019)
		PSY	NO	Garcia et al. (2016)
	Physical abuse	SZ	NO	Aas et al. (2012a)
		PSY	LW	Mansueto et al. (2019)
		PSY	LW	Schalinski et al. (2018)
	Sexual abuse	SZ	LW	Lysaker et al. (2001)
		SZ	NO	Aas et al. (2012a)
		PSY	LW	Mansueto et al. (2019)
		PSY	LW	Schalinski et al. (2018)
	Emotional abuse	PSY	LW	Schalinski et al. (2018)
		PSY	LW	Mansueto et al. (2019)
	Emotional neglect	SZ	NO	Aas et al. (2012a)
		PSY	LW	Mansueto et al. (2019)
	Physical neglect	SZ	LW	Mørkved et al. (2020)
		SZ	NO	Aas et al. (2012a)
		PSY	LW	Mansueto et al. (2019)
		PSY	LW	Üçok et al. (2015)
Attention	General CT/ELS	SZ	NO	Quidé et al. (2018)
		SZ	LW	Ayesa-Arriola et al. (2020)
		FEP	LW	Aas et al. (2011)
		PSY	NO	Garcia et al. (2016)
	Physical abuse	SZ	LW	Aas et al. (2012a)
		PSY	LW	Mansueto et al. (2019)
		PSY	LW	Schalinski et al. (2018)
		PSY	LW	Üçok et al. (2015)
	Sexual abuse	SZ	NO	Aas et al. (2012a)
		PSY	LW	Mansueto et al. (2019)
		PSY	LW	Schalinski et al. (2018)
		PSY	LW	Mansueto et al. (2019)
	Emotional abuse	PSY	LW	Schalinski et al. (2018)
	Emotional neglect	PSY	LW	Schalinski et al. (2018)
		PSY	LW	Mansueto et al. (2019)
		SZ	NO	Aas et al. (2012a)
	Physical neglect	SZ	LW	Mørkved et al. (2020)
		SZ	NO	Aas et al. (2012a)
		PSY	LW	Mansueto et al. (2019)
		PSY	LW	Schalinski et al. (2018)
Processing speed	General CT/ELS	PSY	LW	Üçok et al. (2015)
		PSY	NO	Velikonja et al. (2019)
		PSY	NO	Garcia et al. (2016)
	Sexual abuse	SZ	LW	Lysaker et al. (2001)
Verbal/visual memory	General CT/ELS	SZ	LW	Shannon et al. (2011)
		SZ	LW	Kasznia et al. (2021)
		SZ	NO	Kelly et al. (2016)
		SZ	NO	Quidé et al. (2018)
		FEP	LW	Ayesa-Arriola et al. (2020)
		FEP	LW	Sideli et al. (2014)
		PSY	LW	Velikonja et al. (2019)
		PSY	LW	Mansueto et al. (2018)
	Physical abuse	SZ	NO	Aas et al. (2012a)
	Sexual abuse	SZ	NO	Lysaker et al. (2001)
		SZ	NO	Aas et al. (2012a)
	Emotional neglect	SZ	NO	Aas et al. (2012a)
		PSY	LW	Mansueto et al. (2019)
	Physical neglect	SZ	LW	Li et al. (2017)
		SZ	NO	Aas et al. (2012a)
		PSY	LW	Mansueto et al. (2019)

SZ = patients with schizophrenia spectrum; FEP = patients with first-episode psychosis; PSY = patients with psychosis; LW = impairment; HG = improving; NO = no alterations.

#### 3.4.1. General cognitive ability

The association between CGA and schizophrenic and psychotic patients exposed to CT/ELS was investigated in different studies. In particular, 7 studies showed a correlation of the CT/ELS with a worse general cognitive performance, including language and IQ (Schenkel et al., 2005; Aas et al., 2011, 2012b; McCabe et al., 2012; Schalinski et al., 2018; De-Nardin et al., 2021; Kasznia et al., 2021). On the contrary, 9 studies did not find significant correlations with GCA (Lysaker et al., 2001, 2002; Sideli et al., 2014; Üçok et al., 2015; Garcia et al., 2016; Kelly et al., 2016; Ruby et al., 2017; van Os et al., 2017; Wells et al., 2020).

##### Specific subtypes

When we valued the CT/ELS subtypes, we found that 4 studies (Aas et al., 2012a; Üçok et al., 2015; Kilian et al., 2018; Velikonja et al., 2021) examined the effects of specific types of CT/ELS on general neuropsychological functioning. Noteworthy, no evidence of association was found between physical, sexual, and emotional and GCA; whereas, the results are in contrast for emotional neglect; one study (Kilian et al., 2018) showed that SZ patients were characterized by lower activity in GCA, whereas another study did not find any association (Üçok et al., 2015). Velikonja et al. (2021) tested multiple domains of cognition not only at baseline but also at the onset. In the high-risk psychotic group, there was a trend for better performance in individuals who reported a history of multiple types of CT (abuse and emotional neglect) compared with those with any type of trauma. However, a history of multiple trauma types was not associated with greater cognitive changes in those converters over time, suggesting that there may be different mechanisms that lead to high-risk psychotic states. Aas et al. (2012a) found low GCA performance in SZ patients exposed to physical abuse, whereas Kilian et al. (2018) found this result in patients exposed to physical neglect.

#### 3.4.2. Executive functions

The results obtained assessing patients affected by SZ and psychosis exposed to CT/ELS showed some associations in relation to the EFs performance. Three studies conducted in FEP patients or with high-risk of psychosis (Aas et al., 2011, 2012b; Velikonja et al., 2019) reported that subjects with general CT/ELS had the worst EFs performance. On the contrary, other 3 studies did not find any association between CT/ELS and EFs in patients affected by SZ (Lysaker et al., 2002; Schenkel et al., 2005; Quidé et al., 2018).

##### Specific subtypes

Four studies (Lysaker et al., 2001; Aas et al., 2012a; Üçok et al., 2015; Li et al., 2017) focused their investigation on the role of specific CT/ELS and EFs. Evidence for a relationship between increased levels of physical/sexual abuse and physical neglect and reduced EFs performance was observed. Aas et al. (2012a) showed no association between EFs and sexual/physical abuse and emotional neglect.

#### 3.4.3. Working memory

In 5 studies focusing on SZ and psychosis patients exposed to CT/ELS, 3 studies indicated the presence of reduced WM performance (Aas et al., 2011; Shannon et al., 2011; Velikonja et al., 2019), whereas other 2 studies did not find any associations (Garcia et al., 2016; Quidé et al., 2018).

##### Specific subtypes

The results showed a worse WM performance in patients with physical abuse (Schalinski et al., 2018; Mansueto et al., 2019); sexual abuse (Lysaker et al., 2001; Schalinski et al., 2018; Mansueto et al., 2019), emotional abuse (Schalinski et al., 2018; Mansueto et al., 2019), and physical neglect (Üçok et al., 2015; Mansueto et al., 2019; Mørkved et al., 2020). Aas et al. (2012a) observed no association with CT physical/sexual abuse, emotional and physical neglect and WM.

#### 3.4.4. Attention and processing speed

Studies regarding the attention performance in schizophrenic and psychotic patients exposed to CT/ELS showed the following results. Four findings reported an association between CT/ELS history and reduced attention (Aas et al., 2011; Garcia et al., 2016; Quidé et al., 2018; Ayesa-Arriola et al., 2020), whereas no associations were observed in Garcia et al. (2016) and Quidé et al. (2018). One study showed that psychotic patients had difficulty in PS ability (Üçok et al., 2015).

##### Specific subtypes

We observed that psychotic (Üçok et al., 2015; Schalinski et al., 2018; Mansueto et al., 2019) as well as SZ (Mørkved et al., 2020) patients exposed to physical, sexual, emotional abuse and physical, emotional neglect showed lower attention performance as well as visual–spatial ability and perception. One study found that SZ patients with experience of sexual abuse had difficulty in PS ability (Lysaker et al., 2001).

#### 3.4.5. Verbal and visual memory

In relation to the results performed in SZ and in psychotic patients, 8 studies showed a significant association between the severity of CT/ELS exposure and a worse performance in verbal/visual memory (Shannon et al., 2011; Sideli et al., 2014; Mansueto et al., 2018; Velikonja et al., 2019; Ayesa-Arriola et al., 2020; Kasznia et al., 2021), while 2 studies detected no significant associations (Kelly et al., 2016; Quidé et al., 2018).

##### Specific subtypes

Patients with SZ did not have alterations in memory performance in association with neglect and abuse (Lysaker et al., 2001; Aas et al., 2012a). Two studies found no associations between childhood neglect and immediate recall in patients with psychosis (Li et al., 2017; Mansueto et al., 2019).

### 3.5. CT/ELS exposure and specific cognitive domains in subjects affected by mood, PTSD and GAD disorders

Table 3 shows the main results on the association between CT/ELS with the relative subtypes and specific cognitive profiles in patients affected by MDD, BD, PTSD and GAD disorders.

**Table 3:** Summary of results from studies investigating childhood trauma (CT)/early life stress (ELS) and cognitive functions in mood (major depressive disorder, bipolar disorder), PTSD and GAD disorders.

Cognitive domains	Trauma and subtypes	Mood, PTSD and anxiety disorders	Results	References
General cognitive ability	General CT/ELS	BD	LW	Poletti et al. (2017)
		BD	LW	Martins et al. (2019)
		BD	LW	Bücker et al. (2013)
		MDD	LW	Dannehl et al. (2017)
		MDD	LW	Chakrabarty et al. (2020)
		MDD	LW	Goltermann et al. (2021)
		MDD	NO	Kaczmarczyk et al. (2018)
		MDD	LW	Poletti et al. (2017)
		PTSD	LW	Nakayama et al. (2020)
		PTSD	LW	Burri et al. (2013)
	Physical abuse	BD	LW	Aas et al. (2012a)
		MDD	NO	Chakrabarty et al. (2020)
		PTSD	LW	Nikulina and Widom (2013)
		PTSD	NO	Lantrip et al. (2023)
	Sexual abuse	BD	LW	Savitz et al. (2008)
		BD	NO	Aas et al. (2012a)
		MDD	NO	Chakrabarty et al. (2020)
		PTSD	LW	Nakayama et al. (2020)
		PTSD	NO	Lantrip et al. (2023)
	Emotional abuse	BD	LW	Savitz et al. (2008)
		PTSD	NO	Lantrip et al. (2023)
	Emotional neglect	BD	LW	Savitz et al. (2008)
		PTSD	LW	Lantrip et al. (2023)
	Physical neglect	BD	LW	Jiménez et al. (2017)
		BD	NO	Aas et al. (2012a)
		PTSD	LW	Nikulina and Widom (2013)
		PTSD	NO	Lantrip et al. (2023)
Executive functions	General CT/ELS	BD	NO	Quidé et al. (2018)
		BD	NO	Bücker et al. (2013)
		GAD	LW	Petkus et al. (2018)
		Depressive symptoms/Anxiety	NO	Tjoelker et al. (2022)
	Physical abuse	BD	LW	Aas et al. (2012a)
		MDD	LW	Gould et al. (2012)
		MDD	LW	Dannehl et al. (2017)
		Depressive symptoms/Anxiety	LW	Tjoelker et al. (2022)
	Sexual abuse	BD	LW	Aas et al. (2012a)
		MDD	LW	Gould et al. (2012)
		PTSD	LW	Rivera-Vélez et al. (2014)
	Emotional Abuse	MDD	LW	Gould et al. (2012)
	Emotional neglect	BD	NO	Aas et al. (2012a)
		BD	NO	Hsieh et al. (2020)
		MDD	LW	Gould et al. (2012)
		Depressive symptoms/Anxiety	LW	Tjoelker et al. (2022)
		PTSD	LW	Lantrip et al. (2023)
	Physical neglect	BD	NO	Aas et al. (2012a)
		BD	LW	Hsieh et al. (2020)
		MDD	LW	Gould et al. (2012)
Working memory	General CT/ELS	BD	LW	Bücker et al. (2013)
		BD	NO	Quidé et al. (2018)
		Depressive symptoms/Anxiety	NO	Tjoelker et al. (2022)
		BD	LW	Aas et al. (2012a)
		BD	NO	Aas et al. (2012a)
	Sexual abuse	MDD	LW	Gould et al. (2012)
		MDD	LW	Chakrabarty et al. (2020)
	Emotional neglect	BD	NO	Aas et al. (2012a)
	Physical neglect	BD	NO	Aas et al. (2012a)
Attention	General CT/ELS	BD	LW	Bücker et al. (2013)
		BD	NO	Quidé et al. (2018)
		PTSD	LW	Nikulina and Widom (2013)
		GAD	LW	Petkus et al. (2018)
	Physical abuse	BD	LW	Aas et al. (2012a)
	Sexual abuse	BD	LW	Aas et al. (2012a)
		PTSD	LW	Nikulina and Widom (2013)
	Emotional neglect	BD	NO	Aas et al. (2012a)
		PTSD	LW	Nikulina and Widom (2013)
	Physical neglect	BD	NO	Aas et al. (2012a)
		PTSD	LW	Nikulina and Widom (2013)
Processing speed	General CT/ELS	BD	LW	Poletti et al. (2017)
		BD	NO	Bücker et al. (2013)
		MDD	LW	Saleh et al. (2017)
		MDD	LW	Dannehl et al. (2017)
		MDD	LW	Chakrabarty et al. (2020)
		GAD	LW	Petkus et al. (2018)
	Physical abuse	MDD	LW	Gould et al. (2012)
	Physical neglect	MDD	LW	Gould et al. (2012)
Verbal/visual memory	General CT/ELS	BD	LW	Bücker et al. (2013)
		BD	LW	Savitz et al. (2008)
		BD	NO	Quidé et al. (2018)
		MDD	LW	Dannehl et al. (2017)
		MDD	LW	Chakrabarty et al. (2020)
		Depressive symptoms/Anxiety	NO	Tjoelker et al. (2022)
		PTSD	NO	Pederson et al. (2004)
		PTSD	NO	Stein et al. (1999)
		PTSD	LW	Bremner et al. (2004)
		PTSD	LW	Nakayama et al. (2020)
		PTSD	LW	Bremner et al. (1995)
	Physical abuse	Depressive symptoms/Anxiety	HG	Tjoelker et al. (2022)
		BD	NO	Aas et al. (2012a)
	Sexual abuse	BD	NO	Aas et al. (2012a)
		PTSD	LW	Rivera-Vélez et al. (2014)
	Emotional abuse	MDD	LW	Chakrabarty et al. (2020)
		MDD	LW	Gould et al. (2012)
	Emotional neglect	BD	NO	Aas et al. (2012a)
		MDD	LW	Gould et al. (2012)
	Physical neglect	BD	NO	Aas et al. (2012a)
		MDD	LW	Dannehl et al. (2017)

BD = patients with bipolar disorder; GAD = patients with general anxiety disorder; MDD = patients with major depressive disorder; PTSD = patients with post-traumatic stress disorder; LW = impairment; HG = improving; NO = no alterations.

#### 3.5.1. General cognitive ability

Different studies investigated the association between exposure to CT/ELS and CGA in patients affected by mood, PTSD and GAD disorders. In particular, 6 studies were conducted in BD and/or MDD (Bücker et al., 2013; Dannehl et al., 2017; Poletti et al., 2017; Martins et al., 2019; Chakrabarty et al., 2020; Goltermann et al., 2021), and 2 in PTSD (Burri et al., 2013; Nakayama et al., 2020), showing low GCA performance. One study in MMD (Kaczmarczyk et al., 2018) did not find significant correlations.

##### Specific subtypes

Three studies reported a significant association between increased levels of physical, sexual, and emotional abuse, emotional/physical neglect and reduced GCA performance in patients with BD (Savitz et al., 2008; Aas et al., 2012a; Jiménez et al., 2017). In addition, depressed patients with physical and sexual abuse did not significantly differ in GCA from those without specific CT/ELS (Chakrabarty et al., 2020). Finally, 3 studies were conducted in patients with PTSD, and the results showed that those patients with physical (Nikulina and Widom, 2013) and sexual (Nakayama et al., 2020) abuse, emotional neglect (Lantrip et al., 2023) and physical neglect (Nikulina and Widom, 2013) had poorer cognitive functioning, including language rather than subjects who had not experienced these types of abuse. The remaining studies reported no significant associations.

#### 3.5.2. Executive functions

Here, we described the results concerning the association between EFs abilities and CT/ELS exposure performed in patients affected by mood, PTSD and GAD disorders. One study was conducted in patients with GAD (Petkus et al., 2018), and reported an association between general CT/ELS and worsening in EFs performance. Three studies did not find any associations between CT/ELS and EFs in patients affected by BD (Bücker et al., 2013; Quidé et al., 2018) or depressive symptoms/anxiety (Tjoelker et al., 2022).

##### Specific subtypes

Seven studies (Aas et al., 2012a; Gould et al., 2012; Rivera-Vélez et al., 2014; Dannehl et al., 2017; Hsieh et al., 2020; Tjoelker et al., 2022; Lantrip et al., 2023) found evidence for a relationship between increased levels of physical/sexual abuse and emotional neglect and reduced EFs performance in patients affected by BD, MDD with or without anxiety and PTSD. Specifically for physical and emotional neglect, 2 studies reported low EFs performance in BD and MDD patients (Gould et al., 2012; Hsieh et al., 2020). Aas et al. (2012a) and Hsieh et al. (2020) observed no associations between EFs and emotional and physical neglect.

#### 3.5.3. Working memory

Some evidence was found for patients affected by mood, PTSD and GAD disorders exposed to CT/ELS and WM performance. Two studies showed low WM performance (Aas et al., 2012a; Bücker et al., 2013), whereas the other 2 studies did not detect any association (Quidé et al., 2018; Tjoelker et al., 2022).

##### Specific subtypes

Chakrabarty et al. (2020) presented significant associations between increased levels of sexual abuse and reduction in WM observed in patients affected by MDD. Another study confirmed that sexual abuse was associated with spatial and visual WM deficits in patients with diagnosis of MDD (Gould et al., 2012), whereas Aas et al. (2012a) observed no associations with CT sexual abuse/emotional and physical neglect and WM in BD patients.

#### 3.5.4. Attention and processing speed

Three studies conducted in patients affected by BD, PTSD and GAD showed associations between increased CT/ELS history and reduced attention (Bücker et al., 2013; Nikulina and Widom, 2013; Petkus et al., 2018), whereas no association was observed in Quidé et al. (2018).

Six studies reported a correlation between poor performance in PS features and presence of a general CT/ELS exposure. Out of these six, 3 studies were conducted in patients affected by MDD (Dannehl et al., 2017; Saleh et al., 2017; Chakrabarty et al., 2020), and one in patients with GAD (Petkus et al., 2018). When we considered the BD pathology, one study evidenced a worse performance in PS (Poletti et al., 2017), whereas another one did not find any association (Bücker et al., 2013).

##### Specific subtypes

We observed that BD (Aas et al., 2012a), as well as MDD (Gould et al., 2012) and PTSD (Nikulina and Widom, 2013) patients exposed to physical, sexual abuse and emotional, physical neglect showed lower attention performance as well as visual–spatial ability and perception. No associations were observed in BD patients exposed to emotional and physical neglect (Aas et al., 2012a).

#### 3.5.5. Verbal and visual memory

Eleven studies were performed in patients affected by mood, PTSD and GAD disorders and exposed to CT/ELS and analyzed the potential associations with verbal/visual memory abilities. Two studies were conducted in patients with BD (Savitz et al., 2008; Bücker et al., 2013), 2 in MDD (Dannehl et al., 2017; Chakrabarty et al., 2020), 3 in PTSD (Bremner et al., 1995; Bremner et al., 2004; Nakayama et al., 2020) and all of them reported associations with low verbal/visual memory abilities. On the contrary, 2 studies on PTSD (Stein et al., 1999; Pederson et al., 2004), one in depressive symptoms/anxiety (Tjoelker et al., 2022), and one in BD (Quidé et al., 2018) patients failed to find evidence of a relationship between CT/ELS and memory performance.

##### Specific subtypes

In studies performed in MDD patients that have examined these relationships, physical neglect and emotional abuse or emotional neglect were specifically associated with lower verbal memory (Gould et al., 2012; Dannehl et al., 2017; Chakrabarty et al., 2020). Only one study (Rivera-Vélez et al., 2014) reported associations with sexual abuse in PTSD.

### 3.6. CT/ELS exposure and specific cognitive domains in non-clinical populations

Table 4 summarizes all the available evidence on the associations between specific cognitive domains and general or specific CT/ELS exposure detected in non-clinical populations.

**Table 4:** Summary of results from studies investigating childhood trauma (CT) and early life stress (ELS) and cognitive functions in non-clinical populations.

Cognitive domains	Trauma and subtypes	Results	References
General cognitive ability	General CT/ELS	LW	Perez and Widom (1994)
		LW	D’Amico et al. (2022)
		LW	Lewis et al. (2021)
		NO	Halpin et al. (2022)
		NO	Kobayashi et al. (2020)
		NO	Mert et al. (2016)
	Sexual abuse	LW	Pluck et al. (2011)
		HG	Feeney et al. (2013)
	Emotional neglect	LW	Pluck et al. (2011)
	Physical neglect	LW	Pluck et al. (2011)
		LW	Pesonen et al. (2013)
		LW	Grainger et al. (2020)
Executive functions	General CT/ELS	LW	Daly et al. (2017)
		LW	D’Amico et al. (2022)
		LW	Letkiewicz et al. (2021)
		LW	Lewis et al. (2021)
		NO	Halpin et al. (2022)
		NO	Majer et al. (2010)
	Sexual abuse	HG	Feeney et al. (2013)
	Emotional abuse	LW	Pluck et al. (2011)
	Emotional neglect	LW	Pluck et al. (2011)
	Physical neglect	LW	Pluck et al. (2011)
Working memory	General CT/ELS	LW	Letkiewicz et al. (2021)
		LW	Mark et al. (2019)
	Physical abuse	LW	Roberts et al. (2022)
	Emotional abuse	LW	Majer et al. (2010)
		LW	Roberts et al. (2022)
	Sexual abuse	LW	Roberts et al. (2022)
	Physical neglect	LW	Majer et al. (2010)
		LW	Hawkins et al. (2021)
Attention	General CT	LW	Cromheeke et al. (2014)
		NO	Majer et al. (2010)
	Sexual abuse	NO	Navalta et al. (2006)
Processing speed	General CT/ELS	LW	Korten et al. (2014)
		LW	Lewis et al. (2021)
	Sexual abuse	HG	Feeney et al. (2013)
Verbal/visual memory	General CT/ELS	LW	Goldberg et al. (2013)
		LW	Ritchie et al. (2011)
		NO	Halpin et al. (2022)
		NO	Jelicic et al. (2008)
	Physical abuse	LW	Majer et al. (2010)
	Sexual abuse	LW	Navalta et al. (2006)
		LW	Hawkins et al. (2021)
		NO	Terock et al. (2020)
		HG	Navalta et al. (2006)
		HG	Feeney et al. (2013)
	Physical neglect	LW	Hawkins et al. (2021)
	Emotional neglect	LW	Syal et al. (2014)
		LW	Terock et al. (2020)

LW = impairment; HG = improving; NO = no alterations.

#### 3.6.1. General cognitive ability

Six studies investigated the potential association between exposure to CT/ELS and poorer GCA (Perez and Widom, 1994; Mert et al., 2016; Kobayashi et al., 2020; Lewis et al., 2021; D’Amico et al., 2022; Halpin et al., 2022), and out of them only three studies showed significant associations (Perez and Widom, 1994; Lewis et al., 2021; D’Amico et al., 2022). Other studies did not find any association (Mert et al., 2016; Kobayashi et al., 2020; Halpin et al., 2022) with GCA abilities.

##### Specific subtypes

Results from three studies indicated that higher severity of sexual abuse, emotional neglect, and physical neglect was associated with lower IQ (Pluck et al., 2011; Pesonen et al., 2013; Grainger et al., 2020).

#### 3.6.2. Executive functions

Eight studies evaluated the relationship between EFs and CT/ELS exposure (Majer et al., 2010; Pluck et al., 2011; Feeney et al., 2013; Daly et al., 2017; Letkiewicz et al., 2021; Lewis et al., 2021; D’Amico et al., 2022; Halpin et al., 2022), and results indicated that subjects with a history of childhood maltreatment were showing deficits in cognitive flexibility (inhibition/switching; Daly et al., 2017; Letkiewicz et al., 2021; Lewis et al., 2021; D’Amico et al., 2022). Two studies (Majer et al., 2010; Halpin et al., 2022) did not find significant associations between EFs and a history of CT/ELS.

##### Specific subtypes

The severity of childhood maltreatment (emotional abuse, emotional neglect, physical neglect) was associated with poorer performance in EFs (Pluck et al., 2011). Only one study (Feeney et al., 2013) described the presence of a better performance in this domain in patients exposed to sexual abuse.

#### 3.6.3. Working memory

Although WM is valued on several studies, only 2 reported associations with a history of CT/ELS and deficits in this task (Mark et al., 2019; Letkiewicz et al., 2021).

##### Specific subtypes

Individuals with a history of emotional abuse (Majer et al., 2010; Roberts et al., 2022) or physical neglect (Majer et al., 2010; Hawkins et al., 2021) performed worse in WM. Roberts et al. (2022) found significant associations also between physical and sexual abuse and a lower WM performance.

#### 3.6.4. Attention and processing speed

Three studies examined the relationship between CT/ELS exposure and cognitive domains of attention, and one of them reported deficits in performing attention tasks in subjects with a CT/ELS history as compared to those who had not experienced any trauma (Cromheeke et al., 2014). In contrast, Majer et al. (2010) showed no evidence of association. Moreover, Korten et al. (2014) and Lewis et al. (2021) showed that subjects with a CT history had a worse performance in PS.

##### Specific subtypes

One study, conducted on women victims of childhood sexual violence showed no significant differences in the accuracy and latency scores of attention, as compared to women without history of abuse (Navalta et al., 2006). Feeney et al. (2013) reported a better PS performance in subjects with childhood sexual abuse.

#### 3.6.5. Verbal and visual memory

Subjects with a history of trauma showed more poorly in memory (Ritchie et al., 2011; Goldberg et al., 2013). Conversely, 2 studies did not find significant associations (Jelicic et al., 2008; Halpin et al., 2022).

##### Specific subtypes

Poor performance in visuo-spatial memory was strongly associated with a history of physical abuse (Majer et al., 2010), sexual abuse (Navalta et al., 2006; Hawkins et al., 2021) and physical neglect (Hawkins et al., 2021). However, 2 studies found that subjects who suffered from childhood sexual abuse performed better in memory (Feeney et al., 2013), and verbal memory (Navalta et al., 2006). One study did not find an association between sexual abuse and performance in recall and verbal memory (Terock et al., 2020), whereas 2 studies observed a lower performance in subjects exposed to emotional neglect (Syal et al., 2014; Terock et al., 2020).

### 3.7. Quality and strength of evidence appraisal

We reported in the Supplementary material (Supplementary Tables S4–S6) a detailed description of the methodological quality of the studies as measured on the Newcastle Ottawa Scale. We reported 27 cross-sectional, 28 case–control and 7 cohort-longitudinal studies that matched the criteria for “good” quality. Of note, some methodological limitations/strengths of the included studies were identified. In general, although *a priori* hypotheses were clearly defined and guided all the analyses, the cross-sectional study design did not allow causal associations to be tested robustly. Moreover, although some analyses have found high reliability of self-reports of childhood adversity, their retrospective nature may be influenced by recall bias. All the cohort-longitudinal studies had good follow-up rates. Most of studies utilized validated psychometric instruments and used methods of recruitment which did not limit the generalizability.

### 3.8. Graphical representations of the results

**Figure 2:**
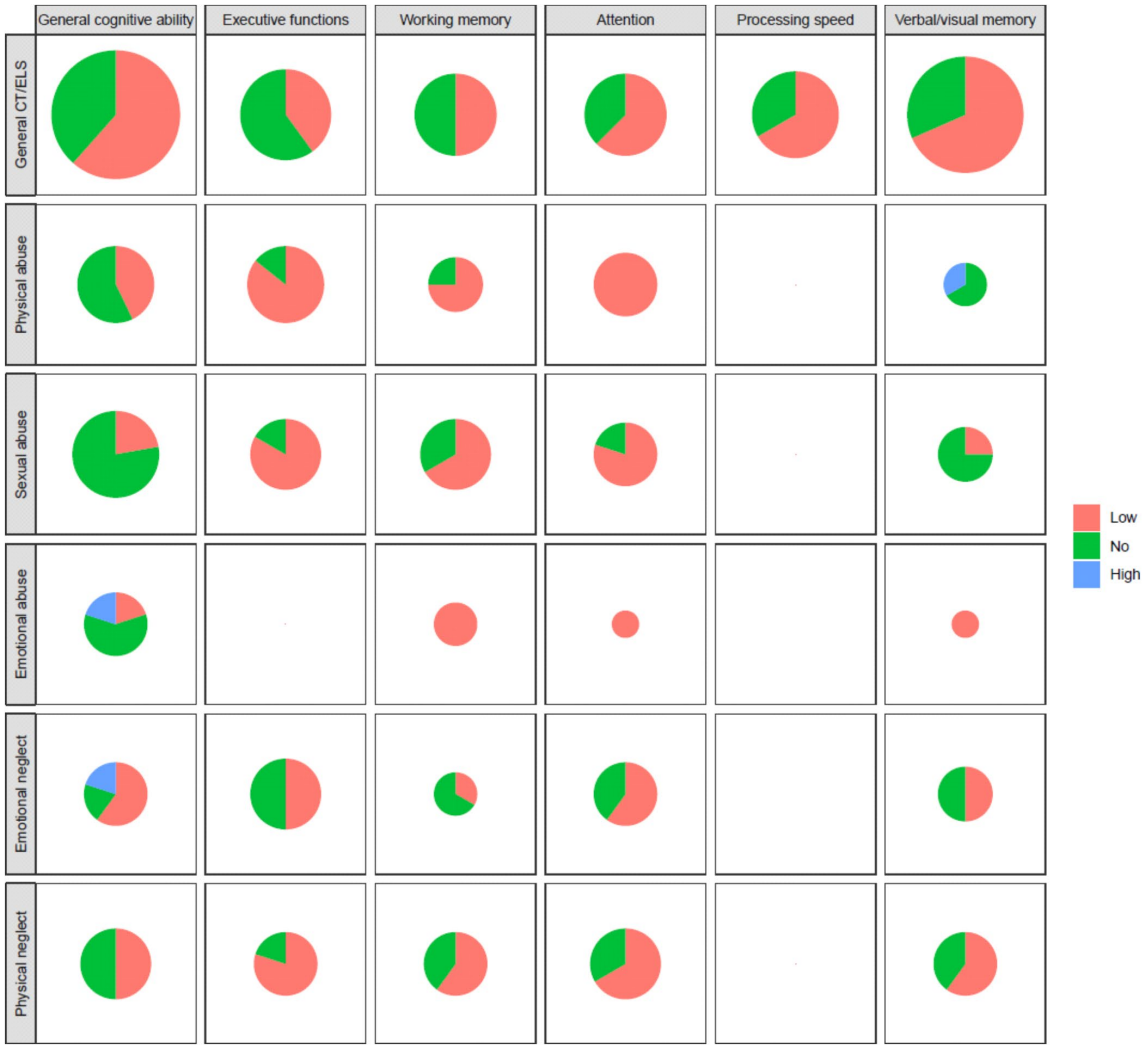
Graphical representations in pie charts performed on psychotic, mood and anxiety disorders exposed to CT/ELs in relation to different cognitive domains (on the columns) and CT/ELS subtypes (on the rows). The size of pie charts area is made proportional to the number of studies considered in each pair of CT type and cognitive ability. Low = impartment; high = improving; no = no alterations.

In Figure 2, the graphical representations showed that CT/ELS exposure had a negative impact on GCA, verbal/visual memory (most of the studies), PS and attention, in patients affected by psychotic, mood and anxiety disorders. In the subtypes analyses, most of the studies revealed significant associations between physical and sexual abuse and physical neglect and EFs, WM and attention performance prevalently in SZ and psychotic disorders; as well as lower performance in verbal/visual memory task in patients exposed to physical neglect. We also observed associations between emotional neglect and deficits in GCA and attention, and poorer WM, attention and verbal/visual memory performance in patients exposed to the emotional abuse.

In the Supplementary Figure S1, we reported the pie charts that refer to SZ and psychosis diagnosis. The most significant results were related to the association between CT/ELS exposure and verbal/visual memory and WM. The worse performance in EFs, WM, visual/verbal memory and attention was detected in physical and sexual abuse, and physical neglect. Associations with WM were observed with emotional abuse, whereas for attention performance, strong evidence was found for emotional abuse and emotional neglect.

In the Supplementary Figure S2, we represented a significant association between general CT/ELS and GCA, verbal/visual memory, PS and attention, in mood disorders, PTSD and GAD. Associations were detected between physical and sexual abuse and physical neglect and EFs, sexual abuse with low attention abilities, as well as emotional neglect and worse EFs/GCA performance, and emotional abuse and verbal/visual memory.

**Figure 3:**
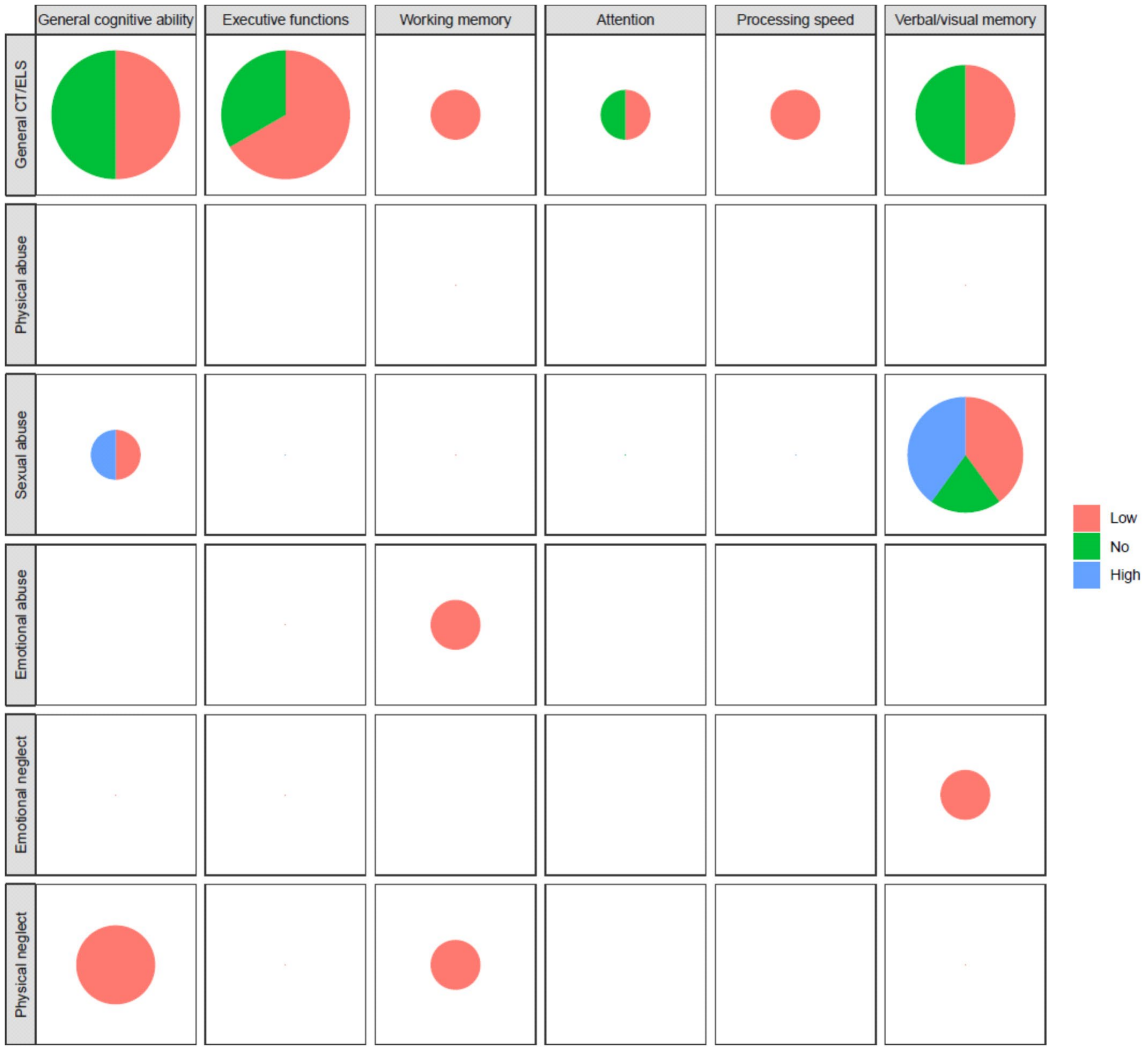
Graphical representations in pie charts performed on non-clinical populations exposed to CT/ELs in relation to different cognitive domains (on the columns) and CT/ELS subtypes (on the rows). The size of pie charts area is made proportional to the number of studies considered in each pair of CT type and cognitive ability. Low = impartment; high = improving; no = no alterations.

Figure 3 showed the graphical representations of the results (pie charts), highlighting the results in non-clinical populations, CT/ELS exposure and specific cognitive domains. Significant associations were detected between CT/ELS and EFs, WM, PS. Emotional abuse was associated with lower WM abilities, whereas emotional neglect to worse performance in verbal/visual memory. Physical neglect was associated with a worse performance in GCA and WM.

### 3.9. The presence of race/ethnicity and income information

In the Supplementary material (Supplementary Tables S1–S3), we described different characteristics of the selected studies including the data related to ethnicity. When we considered the results performed in SZ and psychotic disorders (Supplementary Table S1), 12 studies (Shannon et al., 2011; Aas et al., 2012a; Sideli et al., 2014; Üçok et al., 2015; Garcia et al., 2016; van Os et al., 2017; Mansueto et al., 2018, 2019; Schalinski et al., 2018; Ayesa-Arriola et al., 2020; Mørkved et al., 2020; Kasznia et al., 2021) were carried out in European populations, whereas 6 studies (Lysaker et al., 2001, 2002; Aas et al., 2011, 2012b; Kelly et al., 2016; Velikonja et al., 2019) in mixed populations (White, Black, Afro-American, Asian or others ethnicities), with White ethnicity as the most represented. Three studies were conducted in the United States (Schenkel et al., 2005; Ruby et al., 2017; Velikonja et al., 2021), 3 in Australia (McCabe et al., 2012; Quidé et al., 2018; Wells et al., 2020), one in Brazil (De-Nardin et al., 2021), one in South Africa (Kilian et al., 2018), and one in China (Li et al., 2017).

In the Table S2, we reported the studies conducted in mood, PTSD and GAD disorders. We found 10 studies (Savitz et al., 2008; Aas et al., 2012a; Burri et al., 2013; Dannehl et al., 2017; Jiménez et al., 2017; Poletti et al., 2017; Kaczmarczyk et al., 2018; Goltermann et al., 2021; Tjoelker et al., 2022; Lantrip et al., 2023) performed in European/Caucasian populations, 3 with mixed populations (with the major presence of White; Bremner et al., 1995; Nikulina and Widom, 2013; Saleh et al., 2017), 4 were conducted in the United States (Bremner et al., 2004; Pederson et al., 2004; Gould et al., 2012; Petkus et al., 2018), 3 in Canada (Stein et al., 1999; Bücker et al., 2013; Chakrabarty et al., 2020), one in Australia (Quidé et al., 2018), 2 in Brazil and Latin America (Rivera-Vélez et al., 2014; Martins et al., 2019), and 2 in Asiatic populations (Hsieh et al., 2020; Nakayama et al., 2020).

When we considered the European populations only, we found 5 studies (Shannon et al., 2011; Sideli et al., 2014; Mansueto et al., 2018; Ayesa-Arriola et al., 2020; Kasznia et al., 2021) in SZ and psychotic disorders, that confirmed the association between low verbal/visual memory performance and CT/ELS exposure. In mood, PTSD and GAD, there were 4 studies (Burri et al., 2013; Dannehl et al., 2017; Poletti et al., 2017; Goltermann et al., 2021), that supported associations between lower CGA and CT/ELS exposure.

In all the selected studies, we found and reported data related to duration of education, whereas those related to income, the information was substantially lacking.

As regards non-clinical populations, we described in the Supplementary material (Supplementary Table S3), all the studies that provided information on ethnicity. In particular, 11 studies (Jelicic et al., 2008; Pluck et al., 2011; Ritchie et al., 2011; Feeney et al., 2013; Goldberg et al., 2013; Pesonen et al., 2013; Cromheeke et al., 2014; Korten et al., 2014; Mert et al., 2016; Terock et al., 2020; Lewis et al., 2021) were performed in European populations, and 6 studies on mixed populations (White, Black, Hispanic, Asian, American Indian, or others ethnicities), with the White ethnicity as the most represented (Perez and Widom, 1994; Majer et al., 2010; Daly et al., 2017; Hawkins et al., 2021; D’Amico et al., 2022; Roberts et al., 2022). Four studies were conducted in the United States (Navalta et al., 2006; Mark et al., 2019; Letkiewicz et al., 2021; Halpin et al., 2022), 2 in South Africa (Syal et al., 2014; Kobayashi et al., 2020), and one in Australia (Grainger et al., 2020).

When we considered European populations only, we found that 2 studies showed lower PS activity in association with CT/ELS exposure (Korten et al., 2014; Lewis et al., 2021).

As above, only very few studies reported information regarding the duration of education, whereas no specific details related to the income were reported.

## 4. Discussion

We presented here a systematic review of the literature on the association between adversity during childhood/adolescence, cognitive abilities, and psychiatric symptoms. Due to the high heterogeneity of the topic, we believe that performing a meta-analytic strategy or calculating the effect size with a statistical value could be not sound for our complex aim. High heterogeneity among the studies concerns: 1. different psychiatric pathologies; 2. study designs; 3. different methodological approaches based on patients and controls valued together and/or separately regarding the childhood trauma assessment; 4. different statistical approaches and how the studies reported data; 5. different cognitive battery tests used; 6. different childhood trauma scales used; 7. the fact that the study samples were composed mostly of female participants; 8. different ethnicities (see also Limitations section). For these reasons, we decided to report the results from each study and to represent graphically the data, underling the potential associations between specific CT events and ELS profiles with specific cognitive domains in adults affected by different psychiatric disorders and also in non-clinical populations.

This work supports an association of CT/ELS with specific cognitive features and the development of psychotic, mood and anxiety disorder, also in non-clinical populations. Specifically, CT/ELS exposure has, at several levels, a negative impact on GCA, verbal/visual memory, PS and attention in patients affected by psychotic, mood and anxiety disorders. When considering specific trauma subtypes, most of the studies indicated that physical and sexual abuse, and physical neglect were associated to worse EFs, WM and attention activities. Lower performance in verbal/visual memory task was observed in patients exposed to physical neglect. Furthermore, we observed associations between emotional neglect and deficits in GCA and attention, while poorer WM, attention and verbal/visual memory performance were found in patients exposed to emotional abuse. In non-clinical populations, the study results supported associations between CT/ELS exposure and abnormalities in GCA (for the physical neglect subtype), in EFs, PS, WM (also for emotional abuse and physical neglect) and visual/verbal memory abilities (for emotional neglect). These results are coming from studies performed mainly in European and mixed (prevalently White) populations.

### 4.1. CT/ELS exposure and specific cognitive domains in subjects affected by psychotic, mood and anxiety disorders

The presence of a deficit in GCA after CT/ELS exposure was found exclusively in patients affected by MDD, BD and PTSD (Supplementary Figure S2), whereas no association was observed in SZ and psychotic disorders affected patients (Supplementary Figure S1). A recent meta-analysis supported these findings, as it reported a small negative association with overall cognitive abilities and CT exposure in individuals affected by psychotic disorders, that was significantly weaker than that observed in controls (Vargas et al., 2019). Moreover, significant impairments in PS and in attention were observed in patients with mood, PTSD and anxiety disorders (Supplementary Figure S2), while a significantly deficit in verbal and visual memory was found in all of the considered pathologies. Overall, this confirms the following hypotheses: patients with psychopathology can display deficits in attention, PS and verbal/visual memory (Rock et al., 2014; Scott et al., 2015); and childhood maltreatment represents a major risk factor for the development of psychiatric disorders (Teicher and Samson, 2016; Cross et al., 2017; Clausen et al., 2019), specifically MDD and PTSD.

These results suggest also that the childhood maltreatment leads to a persistent cognitive impairment through the dysregulation of specific biological mechanisms (for instance, dysregulation of HPA axis, Bunea et al., 2017) that, in turn, can increase the risk of developing psychiatric disorders which may further aggravate the cognitive abilities. Exposure to high numbers of CT/ELS events in patients with psychiatric disorders could further contribute to the general worsening in the already impaired cognitive abilities in these individuals.

### 4.2. CT/ELS exposure and specific cognitive domains in non-clinical populations

In the current study, we also support the existence of an association between CT/ELS exposure and abnormalities in EFs, WM and PS abilities in non-clinical populations. Importantly the alterations in PS performance were also observed for psychiatric patients, specifically in those affected by mood, PTSD and GAD (Supplementary Figure S2). The similarity between non-clinical and clinical cohorts suggests that this deficit could not be purely associated with the pathology, rather to the exposure to CT/ELS. Moreover, these results could support a potential role of the cognitive features (EFs, WM, and PS) as potential intervention targets in these populations if exposed to CT/ELS. Finally, the considered non-clinical cohorts were similar to the clinical cohorts in terms of ethnicity and country, as most of the studies was carried out in European cohorts.

### 4.3. Types of exposure

It is well accepted that results on the association of CT/ELS on cognitive performance and on psychopathology suffer from a significant variability in results as different type of traumas and stressors may have a different impact. For this reason, this work wants to further explore this issue by screening the studies based on specific subtypes of CT/ELS and specific domains of cognition, both in clinical and non-clinical populations.

The results obtained indicated the role of physical neglect as a significant predictor of impairment specifically in EFs, WM, attention, and verbal/visual memory performance. This result comes mainly from studies performed on patients suffering from psychotic disorders (Supplementary Figure S1). The involvement of this CT subtype was also observed in non-clinical populations, where an impairment in GCA and WM in association with physical neglect was observed. This could suggest that physical neglect subtype could determine a significant impact on WM impairment, regardless of the pathology (mainly psychosis).

In several studies from Western countries, neglect subtype is the most common type of CT (Hornor, 2014), especially in patients with SZ/psychosis. Indeed, a strong association was observed between this subtype of trauma and hallucinations and delusions in patients affected by FEP/psychotic experiences (Şahin et al., 2013). Moreover, individuals who had experienced physical neglect showed the greatest deficits in cognitive domains (Pears and Fisher, 2005; Nolin and Ethier, 2007). In addition, children neglected during the first 4 years of life had a progressive decline in cognitive functioning, in parallel to significant reductions in their head circumference at 2 and 4 years of age (Strathearn et al., 2001).

Our review also illustrated the existing association between sexual abuse and worse EFs and attention in all the considered pathologies (Figure 2), while associations with WM deficiencies only emerged in presence of psychotic disorders (Supplementary Figure S1). Some studies supported that this subtype can be associated to the onset of a variety of psychiatric disorders (Molnar et al., 2001; Hillberg et al., 2011), and a recent review strongly suggested that sexual abuse is related with attention, in addition to PTSD and BD symptoms (Strathearn et al., 2020).

Furthermore, our graphical representations of the results demonstrated the homogenous relationship between physical abuse and deficits in EFs, WM and attention across all the selected disorders (Figure 2); particularly considering the different pathologies, the abuse most importantly impacted EFs in patients with mood disorders (Supplementary Figure S2) and attention for psychotic disorders (Supplementary Figure S1). Different studies highlighted how this type of trauma is associated with several psychiatric features, such as externalizing behavior problems, delinquency, drug abuse (Strathearn et al., 2020), mood disorders, PTSD, substance abuse disorders, attention deficit hyperactivity disorder (Sugaya et al., 2012), and suicidal ideation (Fuller-Thomson et al., 2012). Jaffee (2017) suggested that exposure to physical abuse may lead to a hypervigilance response to threat, including a disproportionate negative attentional bias, to relatively mild threat cues. Studies have revealed that physically abused children showed selective attention to anger cues (Pollak and Tolley-Schell, 2003), had difficulty disengaging from them (Pollak et al., 2000), and were more likely to misjudge facial cues, such as being angry or fearful. Interestingly, a recent work demonstrated that minors who had been victims of physical abuse showed significant limitations in cognitive flexibility capabilities, attention and inhibitory control, and inability to make decisions, organize and plan the behavior and manage emotions (Moreno-Manso et al., 2022).

In summary, these results emphasize the need of considering prevalently the presence of childhood physical neglect as well as sexual and physical abuse in the pathological underpinnings of psychiatric disorders and that these specific subtypes could be potential predictors of impairment in cognition. Moreover, these findings highlight the potential importance of assessing the EFs, attention and WM measures when we considered studies concerning parent-to-child abuse and neglect. It is also interesting to underline how the combination of various forms of adversity in childhood can have differentiated effects on EFs, for instance. Montoya-Arenas et al. (2022) demonstrated this evidence: early exposure to physical and sexual abuse has a negative impact on the executive skills in adulthood, while some traumatic events related to a long-lasting environment of socio-political violence favor the refinement of executive planning processes, presumably as a mechanism of evolutionary adaptation.

Our work also pointed out how emotional neglect subtype was associated with lower CGA and attention abilities, while emotional abuse exposure was related with worsening in WM, attention and verbal/visual memory in psychiatric patients. However, these results are supported only by few studies if we compared this with the large amount of literature carried on the other subtypes. Regarding the non-clinical populations, the evidence is similar although weaker. History of emotional neglect was associated with lower performance in verbal/visual memory, while emotional abuse was related to poorer WM. Meta-analytic evidence supports the existence of an association between emotional abuse/neglect and higher risks of depression and anxiety (Norman et al., 2012). Emotional neglect may also lead to deficits in emotion recognition and regulation, as well as insensitivity to rewards (Jaffee, 2017), which could potentially influence the social and emotional development. Youth who have been emotionally neglected showed blunted development of the brain’s reward area, the ventral striatum (Hanson et al., 2015), and reduced reward activation may predict the risk for depression (Hanson et al., 2015), addiction (Kim et al., 2017) and other forms of psychopathology. Small-sized studies, mostly based on clinical samples or cohorts referred by Child Protection Services found that emotional abuse was related to poorer spatial WM (Majer et al., 2010), and this was also confirmed by other studies (Augusti and Melinder, 2013; De Bellis et al., 2013; Tran et al., 2017).

In summary, further studies are needed to evaluate the impact of emotional abuse and neglect on cognitive performance in subjects exposed to CT/ELS.

### 4.4. To what extent do our selected studies represent race/ethnicity and income?

Growing evidence suggests that socio-cultural factors (e.g., race/ethnicity and socio-economic status) are among the most influential determinants of health and disease (Hood et al., 2016), and the intrinsic inequities of such factors are strongly correlated with the exposure to CT (Mock and Arai, 2011). Recent work (Goldstein et al., 2021) suggested that the most important duty of public policy, and healthcare programs should be the reinforcement of the notion that children suffer from cumulative adversity across race/ethnicity and income, and that actions have to be done to prevent these adversities or to minimize their effect. Mersky et al. (2021) explored how ACEs prevalence could be influenced by poverty status, race/ethnicity, and gender by using data from the National Longitudinal Study of Adolescent to Adult Health (Add Health). In an unadjusted analysis, the authors reported that ACEs were less prevalent among non-Hispanic Whites than Hispanics, non-Hispanic Blacks and American Indians; whereas greater ACEs exposure was reported by poor subjects. In another study, Assari (2020) demonstrated that the non-Hispanic Black children from high socio-economic status families remained at higher risks of CT exposure, when compared to non-Hispanic White kids. Non-Hispanic Black children were also more likely to develop behavioral problems, mental health issues, and suffer of negative physical health outcomes. Social stratification, racism, segregation, prejudice, and discrimination may be responsible of these results, along with fewer employment opportunities, greater violence, and limited access to healthy food.

Likewise, the measurement of cognitive abilities across different cultural, racial, and ethnic groups has also had a contentious history, with broad political, legal, economic, and ethical repercussions. Although several initiatives have been proposed to overcome these barriers, more collaboration is still necessary to improve the inclusivity when developing and implementing neuropsychological tests. For instance, the European Consortium on Cross-Cultural Neuropsychology (ECCroN), established in 2019, was founded to address these gaps in cross-cultural neuropsychological assessment in Europe (Franzen et al., 2022), and it supports the development, validation, and standardization of widely and cross-culturally applicable tests that consider interindividual variability. Moreover, ECCroN advocates for an improvement of the clinical training of neuropsychologists on culturally sensitive neuropsychological assessment, and for the development and implementation of guidelines for translator-mediated neuropsychological assessments throughout Europe. People from a disadvantaged socio-economic status are at greater risk of cognitive impairment (Letellier et al., 2020; Yang et al., 2020) particularly in the areas of attention, short-term memory, and executive control (Gennetian and Shafir, 2015); and a lower socio-economic status prevents children from developing their cognitive potential, especially in the domains related to language, WM, and EFs (Farah et al., 2006; Merz et al., 2019). Consistently, Letang et al. (2021) reported that socio-economic status mediates the effect of stress on episodic memory, WM, and EFs activities in Black Americans as compared to non-Hispanic White Americans. Social support and social participation have been documented in the literature as further determinants of inequalities in cognitive impairment, therefore, in the recent years, there has been a growing interest in demonstrating the protective influence of higher social support (Kelly et al., 2017), and higher social participation (Gao et al., 2018) on cognitive impairment in older adults.

For what concerns psychiatric pathologies, Dobbins et al. (2023) demonstrated that cognitive impairment was associated with lower CGA impairment score in Black racial group affected by SZ, while the same effect was not observed in White counterpart and these differences were mediated by the level of education. In another study (Czepielewski et al., 2022), the authors highlighted how cognitive abilities in Latin American patients affected by SZ are influenced by demographic and socio-economic factors in low-and middle-income countries. Sedentary behavior, loneliness, and poverty are other important parameters that, in patients with psychiatric disorders, are linked to poor neurocognitive functioning (Thomas et al., 2020). For instance, higher levels of sedentary behavior in SZ patients were associated with lower motor reaction time, and, low levels of overall physical activity were independently associated with worse attention, concentration, and poorer PS (Stubbs et al., 2016). Loneliness was related to several domains of neurocognition in the general population, including GCA, PS, visual memory, and immediate and delayed recall (Shankar et al., 2013; Ayalon and Shiovitz-ezra, 2016).

The studies that we have selected for our review were manly conducted in European/White populations, both in clinical and non-clinical populations. For what concerns the studies related to SZ and psychotic disorders, the results regarding low verbal/visual memory performance and CT/ELS exposure were obtained in European populations. This homogeneity in ethnicity strengthens the comparability of the study cohorts and, thus, the resulting evidence. The same holds for mood disorders, PTSD and GAD and lower CGA. Similarly, in non-clinical populations, the homogeneity in European populations has a critical role in the significance on PS performance. As information regarding the socio-economic status was generally absent in all of the selected studies, it is very hard to delineate conclusions on the relationship between CT/ELS exposure, cognitive domains, psychiatric disorders, healthy populations and socio-cultural and socioeconomical factors, and future studies should consider also these socio-economic variables when testing the association between CT/ELS and cognition.

### 4.5. Limitations

Some recurring, noteworthy limitations were found in the reviewed studies.

First, there are biases in the use of clinical and cognitive tests. The batteries of tests adopted to evaluate ACEs were based on retrospective self-report and unconfirmed self-assessment. Issues regarding the reliability of the self-declared CTs include simple forgetfulness, unawareness, failure to disclose and report mood biases. Nonetheless, all of the included studies used standardized, validated CT batteries which increase the validity of self-reported data (Hardt and Rutter, 2004). In addition, several studies (Brewin et al., 1993; Goodman et al., 1999; Franklin et al., 2002; Fisher et al., 2011) supported that the retrospective memories of ACEs are quite accurate because, according to Krinsley et al. (2003), retrospective falsifications are minimized. A test validated for a standardized list of traumatic experiences should be used to reliably measure trauma exposure and severity. Regarding the evaluation of cognition (CGA, EFs, WM, attention, PS, memory), a plethora of tests is available. While all these tests have their own validation and reliability, biases may arise when comparing the results of different tests.

Second, biological and social factors can be additional variables that can influence cognitive performance also in relation to CT/ELS. Some research has shown that the impact of different types of childhood maltreatment on measures of cognition can be influenced by HPA axis reactivity and genetic variants within HPA axis related genes (i.e., *FKBP5*, (Ferrer et al., 2021) or *IL-6* (King et al., 2021)). Endocrine alterations have also been shown to influence brain structure and function in the general population (McEwen et al., 2015), and in patients with psychosis (Pillinger et al., 2019) and mood disorders (Drevets et al., 2008).

Not all studies conducted in patients affected by psychiatric disorders have reported: (1) the time of onset of the disease, (2) the influence of antipsychotics and antidepressants, (3) medication and the duration of treatments, and that these aspects can influence the cognitive performance (Husa et al., 2017; Prado et al., 2018). Regarding social factors, most of the studies did not take in consideration the effect of recent negative life events, i.e., adult trauma, which could influence the relationship between childhood adversities and cognitive dysfunctions, as individuals with early adverse experiences are more prone to experience traumatic stressors later in life (Majer et al., 2010).

Third, the number of studies available for each diagnostic group is limited. Many of the included studies have focused on patients with SZ, psychosis and BD, whereas only few studies on patients with MDD, PTSD and GAD are available. Moreover, the distribution of participants’ gender was unbalanced across the studies, with most of the study cohorts presenting a majority of females. For instance, when analyzing sex differences on the neurocognitive functioning of maltreated youth, Syal et al. (2014) found a significant effect on adult visuo-spatial cognitive performance in adults with a history of CT. These effects were sex-specific, as associated with improved performance in men and a worse performance in women.

Fourth, some studies adopted a cross-sectional design, which does not elucidate the direction of the causal effects. These cross-sectional studies requested to participants to report events from their past, which generates the risk of recall bias. Regarding this, some authors (Maxwell et al., 2011) have argued that cross-sectional paths (in the absence of reliable estimates regarding prospective relationships within the same variable) provide limited information on prospective paths, whereas others supported the adequacy of cross-sectional data to infer causal links (Pearl, 2009). From our quality assessment, it should be noted that the results of studies performed with a longitudinal design were consistent with the results reported in cross-sectional studies.

Fifth, an additional potential confounder that has implications for data interpretability is the possible exposure of the same individual to different subtypes of trauma, without proper adjustment for co-occurring exposure in the statistical analyses. Vargas et al.’s (2019) study supported this consideration and suggested that promising directions for future research should consider studying whether types of traumas are more impactful than others and evaluating the large possibility of overlap in the exposure to multiple types of traumas. This is particularly relevant as exposure to a specific trauma may increase the likelihood that the same individual will be further exposed to other types of traumas.

Sixth, future studies must carefully take into consideration several forms of trauma, the timing of trauma (the time elapsed between exposure to the traumatic event and testing), as well as the severity/frequency of exposure to trauma, which could help identifying the profiles of individuals at greatest risk for experiencing negative outcomes due to trauma exposure (Vargas et al., 2019).

These factors may also interact and influence the vulnerability to neurocognitive dysfunction. In the reviewed studies, different stress questionnaires based on different assessments of the general CT score, severity and frequency were partly lacking, as discussed by Dauvermann and Donohoe (2019). Thus, studying different CT severity/frequency levels in psychiatric disorders is crucial because the characterization of different levels allows a more detailed interpretation of reduced cognitive function in the context of resilient, susceptible, and compensatory mechanisms, which may necessitate different personalized treatment approaches (Dauvermann and Donohoe, 2019).

## 5. Conclusions and future directions

This is a systematic review that evaluated and support an association of CT/ELS exposure with cognitive performance both in psychiatric and non-clinical populations. Although further studies are needed, PS alterations were shared among the two populations, suggesting an important role of CT/ELS exposure on PS abilities, regardless of the pathology. In addition, this is a systemic review assessing whether specific types of CT/ELS can differentially mediate specific cognitive domains, as well as to what extent the selected studies reported information about ethnicity, income, socioeconomic status and behavior. Physical neglect and physical/sexual abuse could play a crucial role as predictors of impairments in EFs, WM and attention in all the psychiatric conditions that we considered. The same conclusion holds in non-clinical populations for physical neglect and WM; this thus acquires a specific role of physical neglect and WM performance, regardless of the pathology. Further research is needed to confirm the role of emotional abuse/neglect on cognitive functioning in psychiatric and non-clinical populations.

The consistency of these findings suggests that a broad range of cognitive features should be considered when treating patients with traumatic childhood experiences in primary care treatment settings. Specific and targeted educational treatments and personalized interventions in residential care setting should be developed to promote the stimulation, reinforcement, and rehabilitation of the specific cognitive domains that are affected in those individuals who have been exposed to CT/ELS. Future clinical research studies should propose cognitive enhancement programs in individuals with any kind of CT exposure, to reduce the impact of traumatic childhood experiences on future cognitive abilities.

## Author contributions

MR: investigation, writing—original draft, and validation. CS: investigation, writing—original draft, and validation. AC: writing—review and editing, and validation. All authors contributed to the article and approved the submitted version.

## Funding

CS and AC received funding from Ricerca Corrente (Ministry of Health).

## Conflict of interest

The authors declare that the research was conducted in the absence of any commercial or financial relationships that could be construed as a potential conflict of interest.

## Publisher’s note

All claims expressed in this article are solely those of the authors and do not necessarily represent those of their affiliated organizations, or those of the publisher, the editors and the reviewers. Any product that may be evaluated in this article, or claim that may be made by its manufacturer, is not guaranteed or endorsed by the publisher.
