# Supplementary material for: Association of childhood trauma with cognitive domains in adult patients with mental disorders and in non-clinical populations: a systematic review
Source: Front Psychol. 2023 Jun 23;14:1156415. doi: 10.3389/fpsyg.2023.1156415 (PMC10327487; doi:10.3389/fpsyg.2023.1156415)
Supplement: Supplementary file 1 [file Data_Sheet_1.pdf]

Supplementary Material

Effect of childhood trauma on cognitive domains in adult patients with mental disorders and in non-clinical populations: a systematic review

Melissa Rosa[1], Catia Scassellati[1], Annamaria Cattaneo[1, 2]

[1] Laboratory of Biological Psychiatry, IRCCS Istituto Centro San Giovanni di Dio Fatebenefratelli, Brescia, Italy.

[2] Department of Pharmacological and Biomolecular Sciences, University of Milan, Milan, Italy.

***Corresponding Author:**

Dr. Annamaria Cattaneo, PhD

IRCCS Istituto Centro San Giovanni di Dio Fatebenefratelli

Via Pilastroni 4,

25125 Brescia, Italy

Email: acattaneo@fatebenefratelli.eu

annamaria.cattaneo@unimi.it

1 Supplementary Figures and Tables

1.1 Supplementary Tables

Table 1S reported studies on schizophrenia spectrum and other psychotic disorders, **Table 2S** on mood and anxiety disorders: BD, MDD, PTSD, GAD, Table 3S on non-clinical samples.

Tables 4S, 5S, 6S present a detailed description of the methodological quality of the studies as measured on the Newcastle Ottawa Scale.

PRISMA 2020 Checklist a guideline for reporting systematic reviews.

1.2 Supplementary Figures

Fig. 1S. Graphical representations in pie charts performed on psychotic disorders exposed to CT/ELS in relation to different cognitive domains and CT/ELS subtypes. Pie charts area are made proportional to the number of studies considered in each pair of CT type and cognitive ability.

Fig. 2S. Graphical representations in pie charts performed on mood, PTSD and anxiety disorders exposed to CT/ELS in relation to different cognitive domains and CT/ELS subtypes. Pie charts area are made proportional to the number of studies considered in each pair of CT type and cognitive ability.
