# Supplementary material for: Association of childhood trauma with cognitive domains in adult patients with mental disorders and in non-clinical populations: a systematic review
Source: Front Psychol. 2023 Jun 23;14:1156415. doi: 10.3389/fpsyg.2023.1156415 (PMC10327487; doi:10.3389/fpsyg.2023.1156415)
Supplement: Supplementary file 2 [file Data_Sheet_2.pdf]

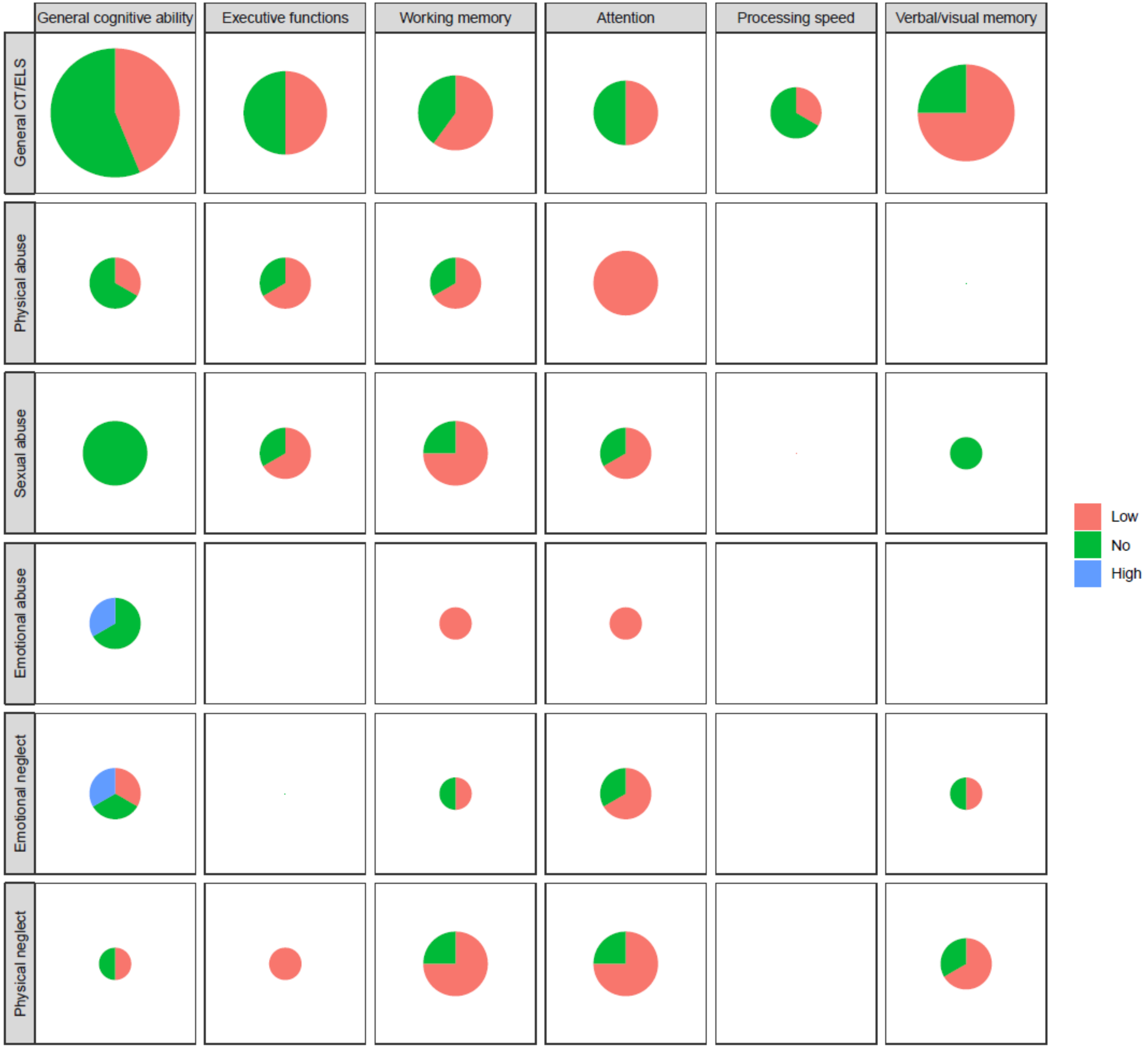

Figure 1S. Graphical representations in pie charts performed on psychotic disorders exposed to CT/ELS in relation to different cognitive domains and CT/ELS subtypes. Pie charts area are made proportional to the number of studies considered in each pair of CT type and cognitive ability.
